# Tree Species Shape Soil Bacterial Community Structure and Function in Temperate Deciduous Forests

**DOI:** 10.3389/fmicb.2019.01519

**Published:** 2019-07-09

**Authors:** Amélie Dukunde, Dominik Schneider, Marcus Schmidt, Edzo Veldkamp, Rolf Daniel

**Affiliations:** ^1^ Göttingen Genomics Laboratory, Department of Genomic and Applied Microbiology, Institute of Microbiology and Genetics, Georg-August University of Göttingen, Göttingen, Germany; ^2^ Soil Science of Tropical and Subtropical Ecosystems, Faculty of Forest Sciences and Forest Ecology, Büsgen Institute, Georg-August University of Göttingen, Göttingen, Germany

**Keywords:** forest soil bacterial community, soil bacteria, Hainich national park, temperate deciduous forest, tree species diversity, soil bacterial diversity, bacterial functional diversity

## Abstract

Amplicon-based analysis of 16S rRNA genes and transcripts was used to assess the effect of tree species composition on soil bacterial community structure and function in a temperate deciduous forest. Samples were collected from mono and mixed stands of *Fagus sylvatica* (beech), *Carpinus betulus* (hornbeam), *Tilia* sp. (lime), and *Quercus* sp. (oak) in spring, summer, and autumn. Soil bacterial community exhibited similar taxonomic composition at total (DNA-based) and potentially active community (RNA-based) level, with fewer taxa present at active community level. Members of *Rhizobiales* dominated at both total and active bacterial community level, followed by members of *Acidobacteriales, Solibacterales*, *Rhodospirillales*, and *Xanthomonadales*. Bacterial communities at total and active community level showed a significant positive correlation with tree species identity (mono stands) and to a lesser extent with tree species richness (mixed stands). Approximately 58 and 64% of indicator operational taxonomic units (OTUs) showed significant association with only one mono stand at total and active community level, respectively, indicating a strong impact of tree species on soil bacterial community composition. Soil C/N ratio, pH, and P content similarly exhibited a significant positive correlation with soil bacterial communities, which was attributed to direct and indirect effects of forest stands. Seasonality was the strongest driver of predicted metabolic functions related to C fixation and degradation, and N metabolism. Carbon and nitrogen metabolic processes were significantly abundant in spring, while C degradation gene abundances increased from summer to autumn, corresponding to increased litterfall and decomposition. The results revealed that in a spatially homogenous forest soil, tree species diversity and richness are dominant drivers of structure and composition in soil bacterial communities.

## Introduction

Forests offer many ecosystem services of ecological and economic significance, provide a diverse, multi-layered habitat for most terrestrial plants and animals, and resources for humans ranging from timber to recreational facilities (Cardenas et al., 2015; Wood et al., 2017). Forests are central hubs of primary productivity and a major sink of carbon, due to the large concentration of trees and other vegetation (Bonan, 2008). Thus, the resulting interplay of ecosystem functions within forests is dynamic and often performed by both biotic and abiotic forest components.

In particular, microorganisms facilitate the exchange of compounds between forest vegetation and forest soils. Forest soils are essential biological matrices in which microbial communities execute key ecosystem functions, such as biogeochemical cycling, through decomposition and mineralization processes mediated by prokaryotes and fungi (Uroz et al., 2016a; Lladó et al., 2017). Factors influencing soil microbial community structure are crucial for predicting how bacteria-mediated processes drive ecosystem responses to environmental changes (Nemergut et al., 2014). Soil bacterial communities are shaped by several edaphic factors, including soil texture and chemistry, as well as biotic factors such as plant roots and associated mycorrhizal activity, aboveground litter, and other decomposing organic matter (Thoms et al., 2010; Lladó et al., 2018).

Compared to grassland or agricultural ecosystems, only a limited number of studies are available on the effects of forest tree species on bacterial community structure and function. These studies revealed complex bacterial community organization that results from dynamic forest resources and processes. Driven by differences in soil pH, beech stands exhibited a higher bacterial diversity compared to spruce stands (Nacke et al., 2011). In mesocosm experiments comprising five mono and mixed species stands, soil bacterial community richness and evenness were strongly influenced by the tree species beech and ash (Pfeiffer et al., 2013). A pan-European study on land-use intensity and microbial co-occurrence indicated that forest soil communities form far richer networks than those of grassland or farm soils (Creamer et al., 2016). Although soil characteristics (particularly pH) have been frequently reported as strong drivers of microbial diversity (Lauber et al., 2008; Kaiser et al., 2016), tree species have been shown to exhibit a stronger impact on community structure than the soil environment (Bonito et al., 2014).

Forest trees exert a stronger influence on soil than other perennial vegetation due to their longevity and the lack of soil management in forests. Over time, changing features, such as forest canopy, root biomass, root exudates, and oxygen and water consumption, change soil temperature, chemistry, porosity, and soil moisture (Augusto et al., 2002, 2015), which in turn affect belowground bacterial communities (Uroz et al., 2016b). Seasonal climate affects tree species-dependent patterns of leaf phenology, leaf litter, and dead wood quality, as well as root biomass development (Goodale et al., 2015; Lladó et al., 2017; Žifčáková et al., 2017). Consequently, belowground carbon (C) allocation, available nitrogen (N), carbon/nitrogen ratio (C/N), and other nutrients of belowground bacterial communities also vary seasonally. This results in selective enrichment of certain taxonomic groups and metabolic processes (Rasche et al., 2011; Yokobe et al., 2018). Tree species such as beech (*Fagus*), spruce (*Picea*), and pine (*Pinus*) have demonstrated stronger acidifying effects on soils due to slow decomposition rates than ash, hornbeam, and lime (Augusto et al., 2002; Uroz et al., 2016b). Soil-acidifying tree species promoted the presence of a high proportion of acidophilic bacterial taxa (Uroz et al., 2016b). Members of *Rhizobiales* and *Burkholderiales* orders were influenced by pH differences arising from root exudates of *Acer*, *Betula*, *Fagus*, and *Quercus* spp. rather than by soil properties or geographic distance (Landesman et al., 2014). The degree of influence on soil bacterial community structure and diversity depends on the tree species, stand type, and spatial arrangement, i.e., mono species or mixed species stands (Klimek et al., 2016; Uroz et al., 2016a). Plant root exudates, including those from trees, produce a highly variable quality and quantity of carbohydrates, organic acids, and other signaling molecules depending on different factors, including tree species, age, as well as environmental and edaphic conditions (Vranova et al., 2013; Urbanová et al., 2015; Zhalnina et al., 2018). Subsequently, root exudates were shown to promote the abundance of taxa beneficial to specific plant species (Zhalnina et al., 2018). Thus, forest trees affect soil bacterial composition and function through direct and indirect processes.

To our knowledge, field studies reporting how different tree species and their spatial arrangement affect soil bacterial communities in deciduous forests are not available. The few studies that focused on how forest stands influence soil bacterial community structure at both entire and active bacterial community level, were conducted in soils with substantial spatial heterogeneity (Chodak et al., 2016; Klimek et al., 2016; Siles and Margesin, 2017; Curd et al., 2018). Although forest trees demonstrated species-dependent selection of specific bacterial taxa, the impact of mono and mixed stands in spatially homogeneous soil, i.e., soil showing uniform physical composition and constant elevation, on soil bacterial community structure was not explored (Uroz et al., 2016b).

The aim of this study was to investigate the influence of tree species on soil bacterial communities in an old-growth broad-leaved temperate forest, the Hainich National Park. We hypothesized that (1) tree species identity drives bacterial community diversity and structure, resulting in taxonomic differences at entire and potentially active bacterial community level, and (2) metabolic functions are also driven by tree species, and follow stand-specific composition. We predicted that (3) pure (mono species) and mixed stands are responsible for changes in soil physicochemical properties, and that bacterial communities in different stands are shaped by season but to a lesser extent than by tree species. We evaluated the hypotheses by comparing 16S rRNA gene and transcript amplicon-derived soil bacterial community data, and functional predictions with soil physicochemical parameters of soils from mono and mixed deciduous tree stands.

## Materials and Methods

### Sample Site Description

**Figure 1:**
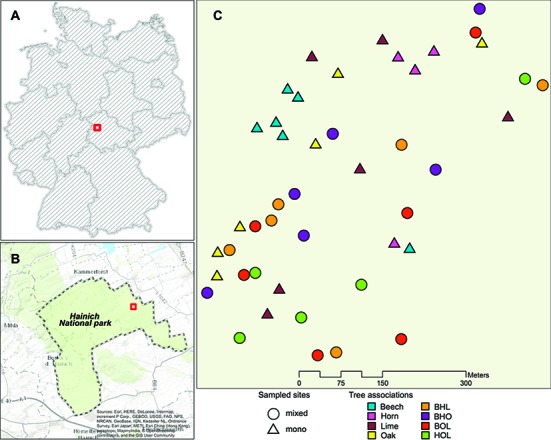
Area of study in the Hainich national park. **(A)** Boundary of the Hainich national park (51°05′37.0 N, 10°30′10.6 E). **(B)** Map of Germany showing location of the Hainich National Park in Thuringia, Germany. **(C)** Sampling area showing distribution of mono stands and mixed stands used for sample collection. Abbreviations stand for the following tree species associations in mixed stands: beech-hornbeam-lime (BHL), beech-oak-hornbeam (BHO), beech-oak-lime (BOL), and hornbeam-oak-lime (HOL). The map was generated by using data from the following data providers: Esri, HERE, DeLorme, Intermap, increment P. Corp., GEBCO, USGS, FAO, NPS, NRCAN, GeoBase, IGN, Kadaster, NL, Ordnance Survey, Esri Japan, METI, Esri China (Hong Kong), swisstopo, Mapmyindia, @OpenStreatMap contributors, and the GIS user community.

The study was conducted over a 25-ha area in the Hainich National Park located in Thuringia, Germany (Figures 1A,B). The park is the largest unmanaged deciduous, broad-leaved forest ecosystem in central Germany, situated near the village of Weberstedt (350 m a.s.l; 51°05′37.0 N, 10°30′10.6 E) (Mölder et al., 2006; Schmidt et al., 2015). The sampling area lies over uniform bedrock of Triassic limestone covered by up to 50 cm of Loess and soil with silt loam/silty clay loam Cambisol (Schmidt et al., 2015). Since the 1960s, the area underwent minor management activity, which was discontinued after its declaration as a national park in 1997 and is therefore described as a natural ecosystem (Kubisch et al., 2015). Core tree species in the mixed-forest ecosystem include the European beech (*Fagus sylvatica*), small-leaved and large-leaved lime (*Tilia cordata* Mill. and *T. platyphyllos* Scop., respectively), oak (*Quercus petraea* and *Q. robur*), and hornbeam (*Carpinus betulus*) (Mölder et al., 2006). Sample sites were selected based on the following criteria: (1) each stand should have a similar surrounding tree species composition displayed, (2) trees must be actively growing and have a well-defined canopy, and (3) homogeneity in soil parameters including color, texture, drainage, and slope of sampling area at initial sampling (Schmidt et al., 2015). Individual stands consisted of four to eight trees, each with an area between 68 and 313 m^2^. Mono-species stands (hereafter referred to as mono stands) comprised beech, hornbeam, lime, and oak, while mixed-species stands (hereafter referred to as mixed stands) consisted of three out of the four tree species: BHL (beech, hornbeam, lime), BHO (beech, hornbeam, oak), BOL (beech, oak, lime), and HOL (hornbeam, oak, lime). Each stand was replicated six times, resulting in a total of 48 plots (Figure 1C).

### Sampling and Environmental Nucleic Acid Isolation

Soil samples were collected in spring (April), summer (July), and autumn (September) of 2012. Two soil cores (10 cm in diameter, 5 cm depth) from the A horizon (topsoil) were extracted from bulk soil at randomly selected polar sites within a plot and pooled to generate a composite sample. Determinations of soil physicochemical properties such as nitrogen (N), carbon (C), phosphorus (P) content, carbon/nitrogen (C/N) ratio and moisture content were performed and described by Schmidt et al. (2015) and are presented in Supplementary Figure S1.

Extraction of environmental DNA and RNA was performed with the MoBio Powersoil DNA isolation kit and RNA Powersoil Total RNA isolation kit, respectively, as recommended by the manufacturer (MoBio Laboratories, Carlsbad, USA). Minor modifications were performed in the extraction of total RNA as follows: after eluting bound RNA from the RNA capture column, a 2.5-fold volume of ethanol and 0.1-fold volume of 3 M sodium acetate (pH 5.2) were added to the RNA, vortexed, and stored at −80°C until required. Solutions were centrifuged at 10,000 × *g* for 1 h (4°C) to recover RNA. Subsequent ethanol/sodium acetate supernatants were discarded, and RNA pellets were dried before suspension in 100 μl distilled water. Residual DNA was removed from extracted RNA by using TURBO DNA-Free kit (Ambion Applied Biosystems, Darmstadt, Germany). Absence of DNA was confirmed according to Wemheuer et al. (2012). Resulting RNA was concentrated with the Rneasy MinElute cleanup kit (Qiagen GmbH, Hilden, Germany). Quantification of DNA and RNA was performed with the NanoDrop ND-1000 UV-Vis spectrophotometer, following the instructions of the manufacturer (Peqlab Biotechnologie GmbH, Erlangen, Germany). Purified RNA (approximately 300–400 ng) was converted to cDNA using SuperScript III reverse transcriptase (Invitrogen, Karlsruhe, Germany) and the bacterial reverse primer V5rev_B 5′-CTATGCGCCTTGCCAGCCCGCTCAG-MID-CCGTCAATTCMTTTGAGT-3′ (Wang and Qian, 2009).

Environmental DNA and cDNA were used as templates to amplify the V3–V5 regions of the 16S rRNA gene by PCR. The 50 μl PCR reaction mixture contained 25 ng of environmental DNA or cDNA as template, 10 μl of 5X Phusion GC buffer, 0.2 μM of each of the four deoxynucleoside triphosphates (dNTPs), 0.4 μM of each primer, 2.5 μl DMSO, 0.15 μl of 25 mM MgCl_2_, and 1 U of Phusion high-fidelity DNA polymerase. The V3–V5 region was amplified with the following set of primers comprising the Roche 454 pyrosequencing adaptors (underlined), a key (TCAG), a unique 10-bp multiplex identifier (MID), and template-specific sequence per sample: the forward primer V3for_B (5′-CGTATCGCCTCCCTCGCGCCATCAG-MID-TACGGRAGGCAGCAG-3′), and reverse primer V5rev_B (see above) from Liu et al. (2007) and Wang and Qian (2009), respectively. Positive and negative control reactions were prepared by using bacterial DNA or substituting water (DNA and RNA-free) instead of the environmental nucleic acids. All PCR reactions were performed in triplicate employing the following thermal cycling parameters for amplification: initial denaturation at 98°C for 5 min, followed by 25 cycles of denaturation at 98°C for 45 s, annealing at 65°C for 45 s and extension at 72°C for 30 s, and a final extension at 72°C for 5 min. Resulting amplicons were analyzed by gel electrophoresis, pooled, and purified with the Qiagen Qiaquick gel extraction kit as recommended by the manufacturer (Qiagen GmbH). Quantification of amplicons was determined by using the Quant-iT dsDNA BR assay kit and Qubit fluorometer as recommended by the manufacturer (Invitrogen GmbH, Karlsruhe, Germany). Amplicon sequencing was performed by the Göttingen Genomics Laboratory with the 454 GS-FLX+ pyrosequencer and titanium chemistry as recommended by the manufacturer (Roche, Mannheim, Germany).

### Processing of 16S rRNA Gene Sequence Data and Statistical Analyses

The resulting 16S rRNA gene and transcript sequences were processed and analyzed with the QIIME (1.9.1) software package (Caporaso et al., 2010) by employing the scheme outlined by Schneider et al. (2015). This involved removal of sequences shorter than 300 bp, containing unresolved nucleotides, exhibiting a low average quality score (<25) or harboring long homopolymers (>8 bp). Forward and reverse primer sequences were removed with the *split_libraries.py* script. Remaining reverse primer sequences, pyrosequencing noise and chimeric sequences, were removed with cutadapt (Martin, 2011), Acacia (Bragg et al., 2012), and UCHIME (Edgar et al., 2011), respectively. Operational taxonomic units (OTUs) were assigned at 97% genetic similarity (species-level) by employing the *pick_open_reference_otus.py* script against the Silva database (release 128) (Quast et al., 2013). Taxonomic classification of OTUs was performed by *parallel_assign_taxonomy_blast.py* script the Silva SSU database release 128. The *filter_otu_table.py* script was used to remove singletons, chloroplast sequences, extrinsic domain OTUs, and unclassified OTUs.

For all statistical tests, a *p* ≤ 0.05 was regarded as significant. Alpha and beta diversity indices and rarefaction curves were calculated with QIIME by using *alpha_rarefaction.py* employing the same level of surveying effort (13,000 randomly selected bacterial sequences per sample). Additional analyses were performed in R (R Development Core Team, 2017). Non-metric multidimensional scaling (NMDS) was performed with the “vegan” package (Oksanen et al., 2019), employing weighted UniFrac distance matrix calculated by *beta_diversity_through_plots.py* (Lozupone et al., 2011). Environmental parameters were fitted on the NMDS with the *envfit* function of the “vegan” package in R (Gergs and Rothhaupt, 2015). A combination of analysis of similarities (ANOSIM) and permutational multivariate analysis of variance (PERMANOVA), implemented in QIIME through *compare_categories.py* were used to improve robustness of multivariate analyses on the effect of tree stands on bacterial community using weighted UniFrac distance matrix (Hartmann et al., 2015). Mantel test was performed using the “vegan” package in R to test correlations between soil physicochemical parameters and soil bacterial communities. Association networks between tree species and OTUs were determined by mapping significant point biserial correlation values calculated by the “indicspecies” package in R (De Cáceres, 2013). Subsequent network visualizations for taxa/tree stand associations were generated with Cytoscape v3.5 by using the “edge-weighted spring embedded layout” algorithm, whereby network edges were weighted by association value (Shannon et al., 2003; Cline et al., 2007). Prediction of functional pathways and corresponding enzymes was performed with Tax4Fun (Aßhauer et al., 2015). Subsequent NMDS and boxplots were calculated with the *vegdist* function in “vegan” and “ggplot2” package, respectively (Gergs and Rothhaupt, 2015; Wickham, 2016).

### Accession Numbers

The 16S rRNA gene and transcript sequences were deposited in the National Center for Biotechnology Information (NCBI) Sequence Read Archive (SRA) under the accession number PRJNA342582.

## Results and Discussion

### Edaphic Properties Reflect Stand-Related Variations

Soil properties displayed significantly different stand-specific variations (*p* < 0.05) except for moisture content (Supplementary Figure S1). Carbon content only differed in hornbeam and the HOL mixed stand. As European forests store large stocks of soil organic carbon (De Vos et al., 2015), soil carbon was not expected to show significant stand-specific concentrations. Among mono stands, lime exhibited the highest mean pH (5.9 ± 0.6). Beech and oak shared a low pH environment (4.6 ± 0.3 and 4.5 ± 0.5, respectively). Mixed stands that included lime displayed higher pH values (BOL 5.4 ± 0.4 and HOL 5.2 ± 0.5) than mixed stands with beech and hornbeam (BHL 5 ± 0.4 and BHO 4.8 ± 0.8).

C/N ratios between 8 and 16 indicate complete breakdown of organic material and, consequently, higher nitrogen availability while phosphorus (P) content, as an essential component for plant growth, indicates organic matter richness and quality (Lauber et al., 2008; Lang et al., 2016). Therefore, we used C/N and P content as indicators of soil fertility (Supplementary Figure S1). Beech and oak mono stands showed high C/N ratios (15.2 ± 0.7 and 16.1 ± 1.3, respectively) compared to lime mono stands (12.4 ± 0.6). Correspondingly, beech and oak mixed stands (BHO and BOL) also exhibited higher C/N ratios compared to lime and hornbeam (BHL) mixed stands (Supplementary Figure S1). Plant available P was consistently higher in soils of hornbeam (585 ± 240 mg/kg) and lime mono stands (536 ± 109 mg/kg) compared to beech (340 ± 41 mg/kg) and oak (335 ± 58 mg/kg). Furthermore, mixed stands with hornbeam and lime (BHL and HOL) exhibited significantly higher P content than mixed stands with beech and oak (BHO and BOL).

Low C/N ratios, high available N and P, as well as high pH, as observed in lime mono stands, promoted tree productivity (Schmidt et al., 2015). In contrast, beech and oak mono stands exhibited higher C/N ratios, low soil available nitrogen and low pH. An explanation could be the decomposition rates of leaf litter. In both mono and mixed stands, lime and hornbeam leaf litter decomposed faster compared to beech and oak leaf litter (Schmidt et al., 2016). Consequently, faster decomposition rates contributed to the observed effect on soil chemistry by a faster release of nutrients (Jacob et al., 2009; Scheibe et al., 2015; Schmidt et al., 2016).

Soil characteristics in mono stands were also recorded in associated mixed stands. This indicates that tree identity and stand type may give rise to distinct microenvironments, whereby mixed stands resulted in intermediate effects in stand productivity compared to mono stands (Schmidt et al., 2015). The results showed a stronger influence of tree species identity over tree diversity on soil characteristics. Our results are supported by similar findings within the Hainich region, which consistently showed a more acidic but potentially nutrient limited soil environment in beech- or oak-dominated stands, compared to lime- or hornbeam-dominated stands (Brunet et al., 1997; Falkengren-Grerup et al., 1998; Salehi et al., 2007; Langenbruch et al., 2012; Berger and Berger, 2014).

### Stand-Specific Patterns in Bacterial Richness and Diversity

Soil bacterial community structure was determined based on 16S rRNA gene and transcript analyses. After processing, quality-filtering and taxonomic clustering at 97% similarity, we recovered 40,385 for entire (DNA-based) and 52,277 OTUs for active (RNA-based) bacterial communities (Supplementary Table S1). The higher number of bacterial OTUs in the active community is in contrast to some other studies of bacterial community diversity in forest (Baldrian et al., 2012; Romanowicz et al., 2016) and grassland soils (Herzog et al., 2015). However, Gill et al. (2017) reported higher RNA-based OTU counts from urban soils, similar to our results. We attribute the different abundances in total and active communities to the detection of very low-abundant but active rare taxa at RNA level.

Diversity indices showed several consistent patterns in mono and mixed tree stands with respect to species richness and evenness, as indicated by Shannon (*H’*), Chao 1, and phylogenetic diversity (PD) (*p* < 0.05; Supplementary Figure S2). Rarefaction curves were close to saturation, and Good’s coverage estimator across all stands remained above 75%, indicating that the sampling effort recovered most of the bacterial diversity.

Among mono stands, lime mono stands showed the highest soil bacterial richness (*H′* 10.5 ± 0.3; PD 169 ± 33) at total and active community level. Similarly, among mixed stands (*H′* 10.4 ± 0.3; PD 170 ± 32) soil bacterial richness was the highest in lime-associated mixed stands (BOL and HOL). Oak mono stands showed significantly lower bacterial richness and diversity (*H′* 9.8 ± 0.4; PD 140 ± 27) compared to other mono stands. We attributed differences in bacterial richness and diversity to tree species-specific effects on the bacterial community. Changes in bulk soil chemistry, i.e., pH differences and nutrient availability, drive bacterial community structure and diversity (Jeanbille et al., 2016; Kaiser et al., 2016). These changes are a direct result of leaf litter and root exudates in soil, which subsequently impact belowground soil bacterial communities (Thoms et al., 2010). In our study, lime mono stands exhibited the highest bacterial richness and diversity and oak the lowest. The favorable physiological conditions (higher pH, low C/N ratio, high exchangeable P) in lime and hornbeam mono stands promoted higher bacterial diversity, compared to the acidic environment of beech and oak mono stands. This is supported by studies in a mixed deciduous forest, which showed similar trends in bacterial community diversity in mono stands and two-species mixed stands of lime, oak and alder (*Alnus* sp.), birch (*Betula* sp.), larch (*Larix* sp.), and spruce (*Picea* sp.) (Šnajdr et al., 2013; Urbanová et al., 2015). Supplementary Figure S3 provides further details on alpha diversity metrics of OTUs at total community (DNA-derived) and potentially active (RNA-derived) levels.

### Tree Species Effect on Bacterial Community Structure

**Figure 2:**
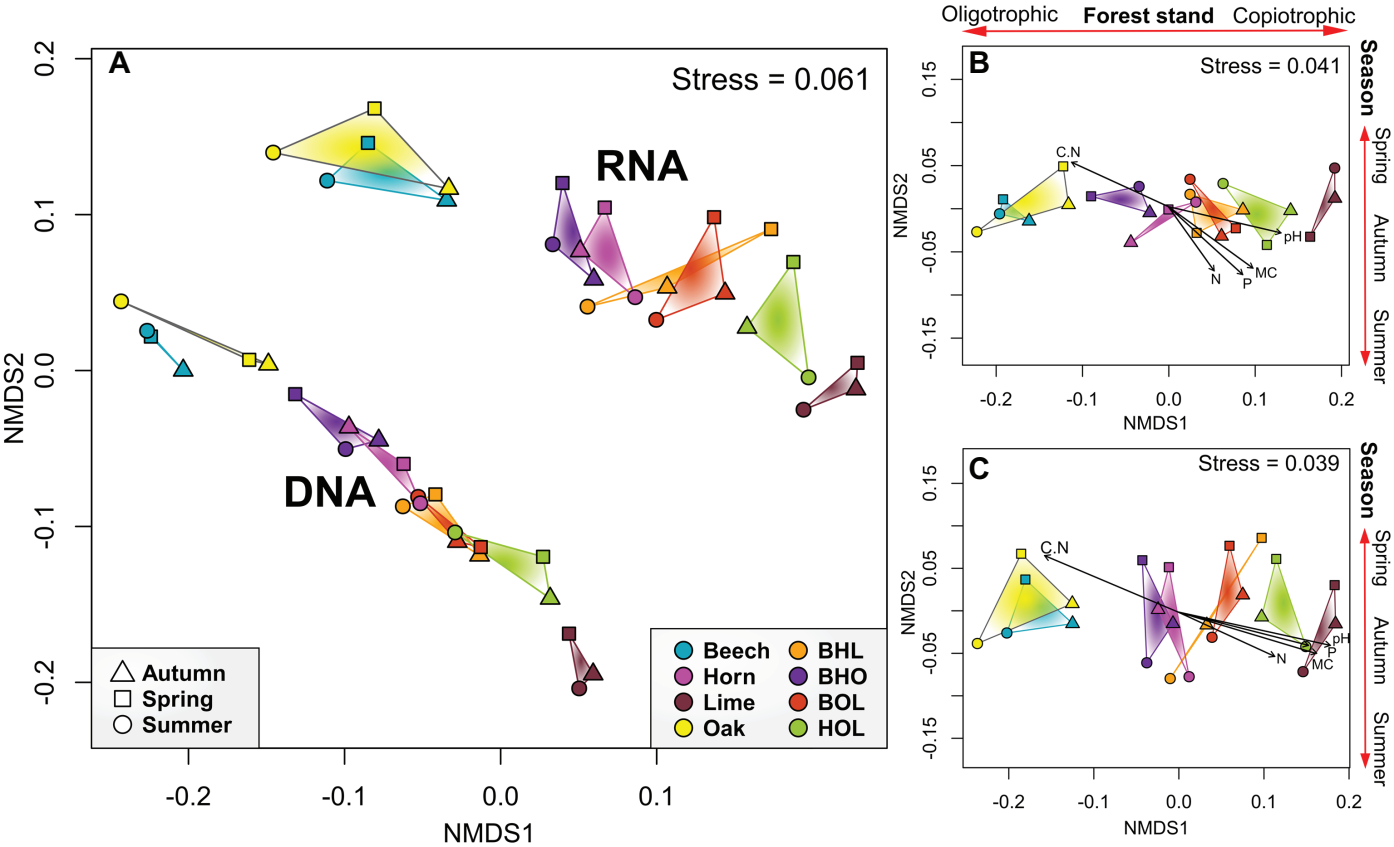
Non-metric multidimensional scaling (NMDS) analysis of soil bacterial communities in mono and mixed stands. **(A)** NMDS plot for total and active community. **(B,C)** NMDS plots for total and active community, respectively, showing the goodness of fit for soil environmental parameters. Ordination employed weighted UniFrac distance matrices of OTUs. Samples are grouped according to season, whereby each data point represents the summarized taxonomic data of six replicate stands. Arrows outside the plot area have no statistical significance and are only a visual aid of observed trends. C/N ratio (C/N), phosphorus content (P), nitrogen content (N), soil moisture (MC), operational taxonomic unit (OTU).

Multivariate analyses confirmed that soil bacterial communities can be differentiated according to tree species (tree species, *p* = 0.001, *r* ^2^ = 0.506) (Figure 2 and Supplementary Table S2). Bacterial communities of each tree species clustered together at total and active community level (template, *p* = 0.001, *r* ^2^ = 0.439). We furthermore detected a pattern in which communities in mono stands separated from those in mixed stands (stand type, *p* = 0.043, *r* ^2^ = 0.068). Exceptions were bacterial communities in hornbeam mono stands, which grouped with mixed stands. Results of the NMDS were supported by ANOSIM and PERMANOVA tests, which indicated that tree species was a strong driver of bacterial community structure at total and active community level (*p* = 0.001, ANOSIM and PERMANOVA; Supplementary Table S2). Stand type also exhibited a strong influence on community structure at the potentially active community level (*p* = 0.002 and *p* = 0.044, ANOSIM and PERMANOVA, respectively). The results supported the trends observed in alpha diversity analyses, in which bacterial richness and evenness show stand-specific variation. Thus, these results confirm hypothesis (1), that tree species drive bacterial community composition.

Seasonal effects in our data were less pronounced (season, *p* = 0.858, *r* ^2^ = 0.0142) (Figure 2A, Supplementary Table S2), although a loose clustering of communities was observed (Figures 2B,C). ANOSIM and PERMANOVA analyses indicated that season was significantly correlated with bacterial communities at total community level (*p* = 0.464 and 0.529, respectively), but ANOSIM showed a significant seasonal correlation at active community level (*p* = 0.038). Although temporal seasonality is an important factor in temperate deciduous forests, which influences plant phenology, and subsequent root exudation and nutrient uptake processes (Oh et al., 2012), we did not observe a strong link between season and bacterial taxonomic composition in this study. Correspondingly, a warming experiment of a temperate forest soil only showed a shift in bacterial community composition after 20 years and only in the organic horizon (DeAngelis et al., 2015). Seasonal effects on soil bacteria at active community level can be explained by small changes driven by “conditionally rare taxa” (CRT), which periodically increase environmental during favorable conditions (Shade et al., 2014). Moreover, CRT may maintain low abundance within the environment but during fluctuating conditions, as during seasonal changes, react by rapidly increasing the abundance of the ribosomal content (Hausmann et al., 2016). The active bacterial community is more sensitive to environmental disturbance than the total community (Pochon et al., 2017), as environmental RNA almost exclusively originates from viable organisms (Moran et al., 2013; Pochon et al., 2017). These effects are likely to produce distinct seasonal communities at active community level.

Edaphic parameters also contributed significantly in shaping the bacterial community (*p* = 0.001; Figures 2B,C; Supplementary Table S2). The Mantel statistic showed a significant positive correlation of soil parameters with bacterial communities (Supplementary Table S2). In particular, a strong influence of soil pH, P, N, and soil moisture was recorded in lime and hornbeam mono stands, and in the corresponding BHL, BOL, and HOL mixed stands compared to the other stands. Bacterial communities in beech and oak mono stands and the BHO mixed stand responded to an increase in C/N ratio. Nutrient availability in soil has been linked to soil bacterial community structure (Bergkemper et al., 2016). Our results show that the nutrient-rich soils of lime and hornbeam mono stands possess bacterial community structures, which are distinct from those in comparatively nutrient-reduced soils in beech and oak mono stands.

Rather than tree species richness (mixed stands), tree species identity (mono stands) contributes to bacterial diversity and structure, as tree species showed a stronger positive correlation with bacterial communities than stand type (tree species *r* ^2^ = 0.506; stand type *r* ^2^ = 0.068). A study in a mixed deciduous forest demonstrated a similar correlation of microbial diversity with tree species identity, while species richness influenced microbial composition (Khlifa et al., 2017). Plant-specific traits such as fine root biomass and density, and leaf litter quality contributed to nutrient availability in soil (Thoms et al., 2010). Beech stands were shown to have higher fine root biomass than hornbeam or lime (Jacob et al., 2013), which could have a stronger impact on bacterial communities than other mono stands. However, beech showed no overyielding of root biomass in mixed stands (Langenbruch et al., 2012; Jacob et al., 2013). This explains that the impact of mixed stands on bacterial communities is often similar to the constituent mono stands, as little competition exists in fine roots. Furthermore, a significant change in soil bacterial community was observed with increasing horizontal distance from trees and demonstrated shifts in nutrient availability, such as decreasing concentrations of organic nitrogen compounds (Nacke et al., 2016). These changes correspond to a decrease in species-dependent root biomass density and subsequent nutrient availability.

Root exudation is plant-species dependent (Zhalnina et al., 2018). The release of exudates into the soil can be direct or regulated further through interactions of arbuscular mycorrhizal or ectomycorrhizal fungi with plant roots (Meier et al., 2013; Kaiser et al., 2015; Canarini et al., 2019). Current understanding of the transfer of root exudates from the rhizosphere to the bulk soil is still poorly understood; however, evidence suggests that nutrient transfer by ectomycorrhiza into the soil results in rapid changes in nutrient conditions of the surrounding soil (Gorka et al., 2019). All tree species in our study show ectomycorrhizal colonization (Liese et al., 2018), and the degree of mycorrhiza influence on bacterial community structure is also likely dependent on tree species.

### General Patterns in Bacterial Community Composition and Structure

**Figure 3:**
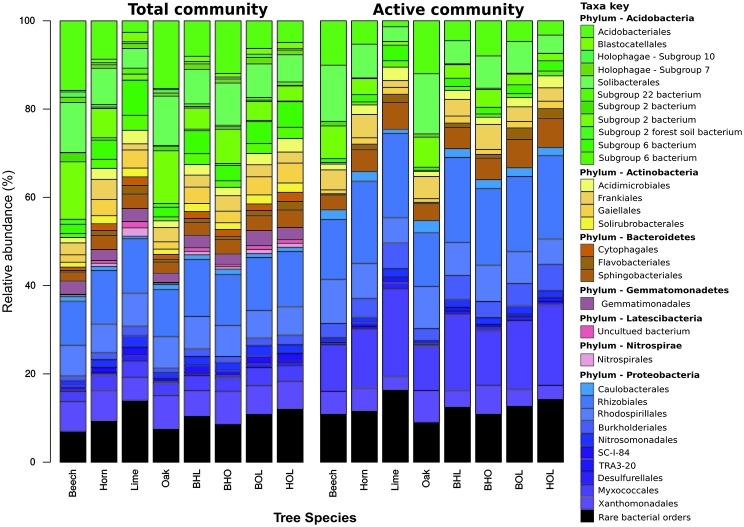
Mean relative abundance of soil bacterial communities in mono and mixed stands. Results show bacterial community composition at order level, whereby each stand represents the average abundance of *n* = 3 seasons (spring, summer, and autumn). Total and potentially active taxa were derived from environmental DNA and RNA, respectively. Taxa with a relative abundance less than 1% across all stands were grouped into “rare bacterial orders.” Abbreviations for mixed stands: beech-hornbeam-lime (BHL), beech-hornbeam-oak (BHO), beech-oak-lime (BOL), hornbeam-oak-lime (HOL).

The entire dataset comprised 40 bacterial phyla, 155 classes, 385 orders, 704 families, and 1,552 genera. Dominant phyla (relative abundance > 1%) across the entire dataset comprised *Acidobacteria*, *Proteobacteria, Actinobacteria*, *Bacteroidetes*, *Gemmatimonadetes, Chloroflexi*, *Latescimicrobia*, and *Nitrospirae* (Figure 3). *Acidobacteria* dominated at total bacterial community level (37.2% at DNA level and 23.2% at RNA level) and *Proteobacteria* at active community level (36.8% at DNA level and 56% at RNA level). Generally, soils contain a few dominant bacterial phyla, mainly *Acidobacteria*, *Actinobacteria*, *Bacteroidetes*, and *Proteobacteria* (Fierer et al., 2012) that have been found consistently in both cultivated and natural environments (Shi et al., 2011; Shange et al., 2012; Ferrenberg et al., 2013; Shen et al., 2014; Schneider et al., 2015).

Bacterial community composition was stand-specific (Supplementary Figures S4, S5) and showed dependence on pH and nutrient availability (Supplementary Figure S1). Copiotrophic members, such as *Alpha*- and *Betaproteobacteria*, *Actinobacteria*, and *Bacteroidetes*, are adapted to nutrient-rich environments, as found in the soils of lime stands. In contrast, members of *Acidobacteria* are recognized as oligotrophs, which are adapted to nutrient-limiting conditions (Fierer et al., 2012; Shange et al., 2012; Koyama et al., 2014). Beech stands, which have been frequently associated with nutrient-limited acidic soils, favored acidobacterial groups (Uroz et al., 2011; Jeanbille et al., 2016; Lladó et al., 2016; Kielak et al., 2016b; Colin et al., 2017).

*Proteobacteria* were evenly distributed across all sites with slight differences between mono stands and mixed stands, at total community level (Figure 3 and Supplementary Table S3). The *Rhizobiales* order within *Alphaproteobacteria* were more abundant in lime and hornbeam mono stands at total community and active community level than in beech and oak mono stands (Figure 3 and Supplementary Table S3). A similar trend was observed in the mixed stands BHL and HOL compared to BHO and BOL. At genus level, we observed that these differences were due to high abundances of unidentified members of the *Xanthobacteraceae* family, *Bradyrhizobium*, *Rhizomicrobium*, and *Variibacter. Rhizobiales* are broadly associated with nitrogen fixation, plant pathogenicity, and organic matter decomposition (Carvalho et al., 2010). The *Rhodospirillales* order, which comprised mostly uncultured groups at genus level, showed high abundance in soils of beech and lime mono stands, due to the presence of the *Acetobacteraceae* family, in beech mono stands (up to 4.7% at DNA and RNA level) and uncultured *Rhodospirillum* DA 111 in beech and lime mono stands (up to 11.3% at DNA and RNA level). *Acetobacteraceae* contains some acidophilic genera of acetic acid bacteria (AAB), which are adapted to acidic environments (Mamlouk and Gullo, 2013), and explains the higher abundance in acidic stands. The *Reyranella* genus was enriched in lime mono stands, which was consistent with previous reports of forest soil bacterial communities (Felske et al., 1998; Kim et al., 2013).

*Betaproteobacteria* were represented by *Burkholderiales* (unidentified *Comamonadaceae* genus, *Variovorax* and *Rhizobacter*) and *Nitrosomonadales* (unidentified *Nitrosomonadaceae* genus and *Nitrosospira*) (Supplementary Figures S4, S5; Supplementary Table S3). At total and active community level, *Burkholderiales* and *Nitrosomonadales* were more abundant in lime and hornbeam mono stands (6.8 and 9.5%, respectively) compared to beech and oak mono stands (3.8 and 4.5%, respectively). A similar composition was also found in the mixed stands BHL and HOL compared to mixed stands BHO and BOL. Both orders form part of the nitrogen-fixing bacterial community in forests soils and participate in symbiotic relationships with plants (Cherobaeva et al., 2011; Tkacz and Poole, 2015).

*Myxococcales* and *Desulfurellales* orders dominated within *Deltaproteobacteria* (Figure 3). *Haliangium* was the most abundant genus within *Myxococcales* at total community level (1.3%) and active community level (6%), followed by *Sorangium*. Both genera are commonly found in soils, but only members of *Sorangium* have been isolated from soil (Dawid, 2000; Fudou et al., 2002). Members of both genera exhibit a capacity for producing secondary metabolites with potential pharmaceutical use (Fudou et al., 2001; Li et al., 2014). The *Desulfurellales* consisted mainly of *Desulfurellaceae* family members. *Desulfurellaceae* were more abundant in lime and hornbeam mono stands compared to beech or oak mono stands at total and active community level (Supplementary Table S3). *Desulfurellaceae* are obligate sulfur-metabolizing thermophiles that contribute to the sulfur cycle (Flores et al., 2012; Wang et al., 2016). Their presence in forest soils is not widely reported but one study has reported *Desulfurellaceae* in farm soils (Wang et al., 2016).

The *Gammaproteobacteria* consisted primarily of *Xanthomonadales* (*Rhodanobacter*, *Acidibacter*, and an unidentified genus). Members of *Xanthomonadales*, including *Rhodanobacter*, have been reported to prefer soil environments with low pH and high C/N ratios, which promote efficient denitrification (van den Heuvel et al., 2010; Green et al., 2012; Prakash et al., 2012). Correspondingly, *Xanthomonadales* were enriched at total and active community in beech (6.9 and 5.1%, respectively) and oak mono stands (7.7 and 7.3%, respectively) compared to lime mono stands (5.2 and 3%, respectively).

The composition of *Acidobacteria* varied considerably across forest stands, through several unidentified subgroups (Supplementary Figures S4, S5). *Acidobacteriales*, *Solibacterales*, and subgroup 2 were more abundant in beech and oak mono stands compared to lime and hornbeam mono stands (Supplementary Figures S4, S5; Supplementary Tables S3 and S4). In contrast, subgroup 6 showed higher relative abundance in lime and hornbeam mono stands (19% at DNA level and 7.3% at RNA level) than in beech and hornbeam mono stands (6.3% at DNA level and 2.2% at RNA level). In mixed stands, *Acidobacteriales*, *Solibacterales*, and subgroup 2 exhibited similar trends as in mono stands and were more abundant in beech and oak mixed stands, BHO and BOL. Subgroup 6 showed higher abundance in lime and hornbeam mixed stands BHL and HOL, at total and active community level. *Acidobacteria* correlate negatively with pH and nutrient availability (Sait et al., 2006; Clivot et al., 2012; Miyashita, 2015; Kielak et al., 2016a). Acidobacterial groups with copiotrophic lifestyles, which includes subgroup 6, were enriched in more neutral, nutrient-rich environments (Naether et al., 2012; Huber et al., 2016), which confirms our results.

*Actinobacteria*, which includes plant pathogens and members capable of producing secondary metabolites (Ventura et al., 2007; Barka et al., 2016), was mainly represented by the *Gaiellales*, *Frankiales*, *Acidimicrobiales*, and *Solirubrobacterales* orders (Supplementary Tables S3 and S4). *Frankiales* abundance increased in the active community (4%) compared to the total community (2.9%). Many dominant genera within these orders were unclassified but we identified *Acidothermus* within *Frankiales*. *Acidothermus* showed lower abundance in lime mono stands compared to beech, hornbeam, and oak, at total and potentially active community levels (Supplementary Tables S3 and S4). *Acidothermus* was previously recorded in high relative abundance (11.5% at DNA level) from a natural forest (Kim et al., 2015). Additionally, the only cultured species, *Acidothermus cellulolyticus*, was isolated from an acidic hot spring, which points to an acidophilic lifestyle, as shown by our study (Mohagheghi et al., 1986; Barabote et al., 2009).

Representatives of *Bacteroidetes* belonged predominantly to *Cytophagaceae* (*Cytophagales*), *Flavobacteriaceae* (*Flavobacteriales*), and *Chitinophagaceae* (*Sphingobacteriales*) families, which showed higher abundance in lime and hornbeam mono stands than in beech and oak mono stands (Supplementary Figures S4, S5; Supplementary Tables S3, S4). The genus *Flavobacterium* within *Flavobacteriaceae*, is a common soil inhabitant, and was more enriched at active community level. *Flavobacterium* was more abundant in lime and hornbeam mono stands (2.8% at DNA level and 2.9% at RNA level) than in beech and oak mono stands (0.78% at DNA level and 0.56% at RNA level). Species of *Flavobacterium* have been reported in plant root associations and deadwood, and harbor broad physiological capabilities including lignolytic activity (Dilly et al., 2000; Kolton et al., 2013; Deshmukh and Sao, 2015; Hoppe et al., 2015).

### Taxa-Habitat Association Patterns

**Figure 4:**
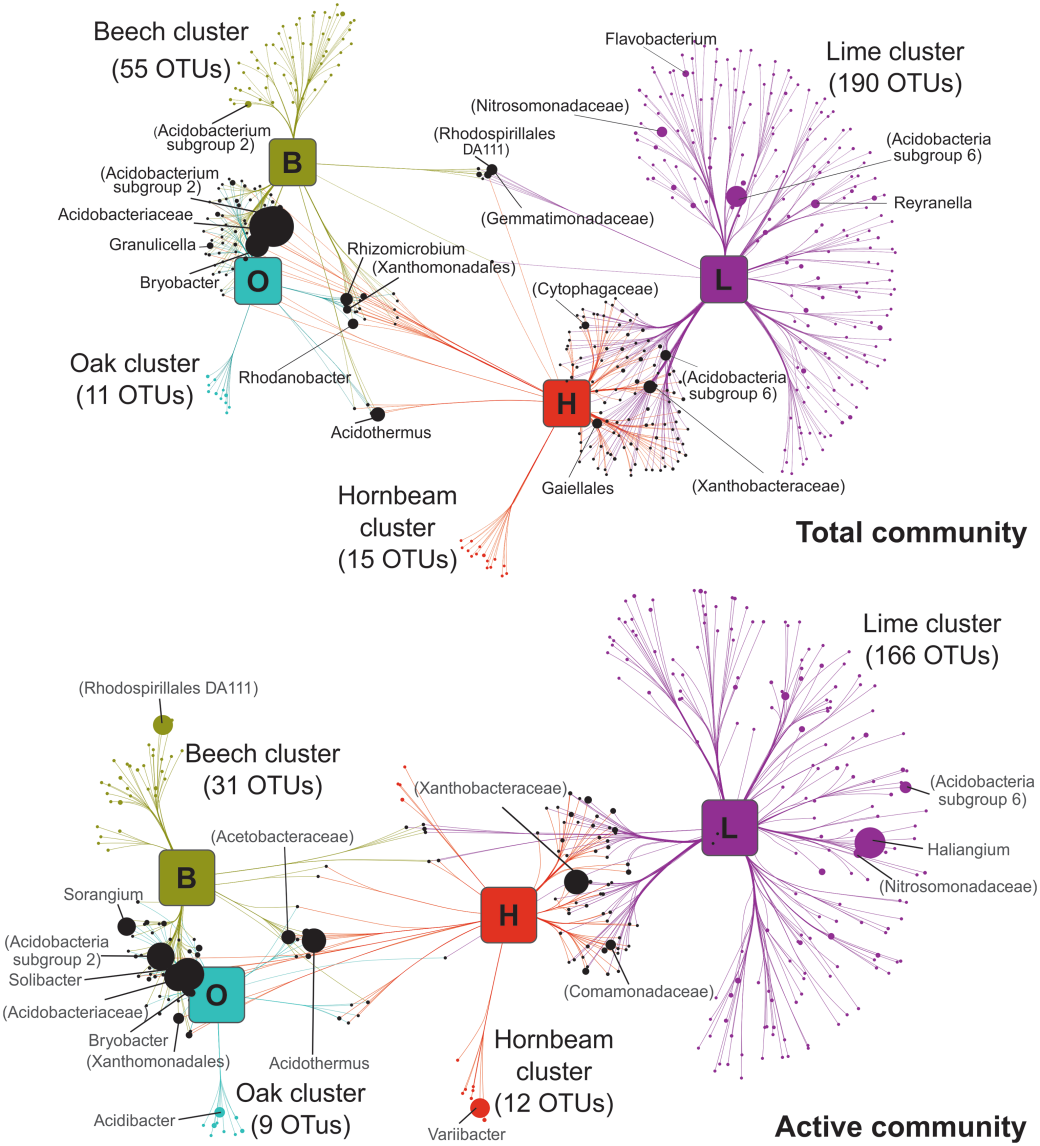
Association networks between soil bacterial communities (genus level) and mono stands. Source nodes (rounded squares) represent mono species tree stands and edges represent associations between stands and bacterial OTUs (circles, target nodes). Edges are colored according to the source tree species and the length of edges is weighted according to association strength. Unique clusters, which associate with one tree species, consist of nodes colored as the corresponding stand. Numbers of OTUs making up respective unique clusters are given in brackets. Black circles represent OTUs with significant cross association between two or more plots. Target node sizes represent mean relative abundance of OTUs across all mono plots. Data only represents OTUs that showed significant positive association with tree species (*p* ≤ 0.05). In the case that the genus could not be assigned, the taxonomic name at the highest determined taxonomic resolution is given in parenthesis. For ease of visualization, edges were bundled together, with a stress value of 3. Abbreviations: beech (B), hornbeam (H), lime (L), oak (O), and operational taxonomic unit (OTU).

Bipartite association networks provided insight into bacterial taxa that potentially drive the observed community structures across tree stands (Figure 4; Supplementary Figure S6). Networks were constructed from OTUs showing significant positive associations to specific stands or a combination of stands. We detected 466 and 348 indicator OTUs at total and active community level, respectively. The correlation-based network strongly mirrors the pattern of forest stands recorded during NMDS analysis (Figure 2). Shorter cross edges between beech and oak stands compared to hornbeam or lime stands indicate that communities in beech and oak stands are more closely associated with tree species than those associated with hornbeam or lime stands. No significant positive associations were observed between bacterial genera in oak and lime mono stands.

Unique clusters representing OTUs associated significantly with only one mono stand, accounted for 58 and 64% of all network OTUs at total and active community level, respectively. The higher number of genera that define unique clusters in lime stands in the total (191) and active (169) community arises from the high bacterial diversity observed in lime stands compared to hornbeam, beech, and oak stands. Unique clusters in mixed stand were less common and instead, OTUs formed cross associations with two or more mixed stands (Supplementary Figure S6). This provides further evidence that bacterial communities in mixed stands are composed of members associated with corresponding mono stands and illustrates the importance of tree species identity over tree species richness in shaping soil bacterial community.

Genera in unique clusters belonged to dominant phyla and orders described in this work. At total community level, we identified unclassified OTUs from *Acidobacteria* subgroup 6, *Nitrosomonadacea* and *Reyranella* genus in lime mono stands. Any overlap of significant bacterial cross-associations between beech and oak, and hornbeam and lime mono stands was provided by a few genera and comprise, among others, *Rhodanobacter*, *Rhizomicrobium*, *Acidothermus*, *Bryobacter*, *Granulicella*, members of *Gemmatimonadacea*, and members of *Cytophagaceae*. This pattern is similarly reflected in the active community but with fewer genera. Analyses of soil environments have revealed shared taxonomic groups, but only a few genera participate in distinguishing one soil habitat from another (Hartmann et al., 2015; Rime et al., 2016). A study on microbial community conversion between organic and conventional farming showed that only 12% of bacterial OTUs constituted the management-specific community, with 49% showing significant unique association to a specific management type (Hartmann et al., 2015). Our results follow this trend, as only a small fraction of total OTUs define the difference between soil bacterial communities of different tree stands.

### Bacterial Functional Profiles Across Forest Stands

The 16S rRNA transcript-based communities were used to predict active metabolic processes in forest soil. It has to be noted that rRNA abundance is only a qualitative index for activity but not direct measure of activity (Blazewicz et al., 2013). Thus, the predicted functional profile does not necessarily reflect the direct activity of the studied organisms. Additionally, OTUs derived from unknown taxa limit functional predictions. Nevertheless, it has been shown for bacterioplankton and soil bacterial communities, as well as for communities in other environments that 16S rRNA-derived functional prediction are in good agreement with those derived from direct sequencing of corresponding metagenomes and metatranscriptomes (Aßhauer et al., 2015; Kaiser et al., 2016; Wemheuer et al., 2017).

**Figure 5:**
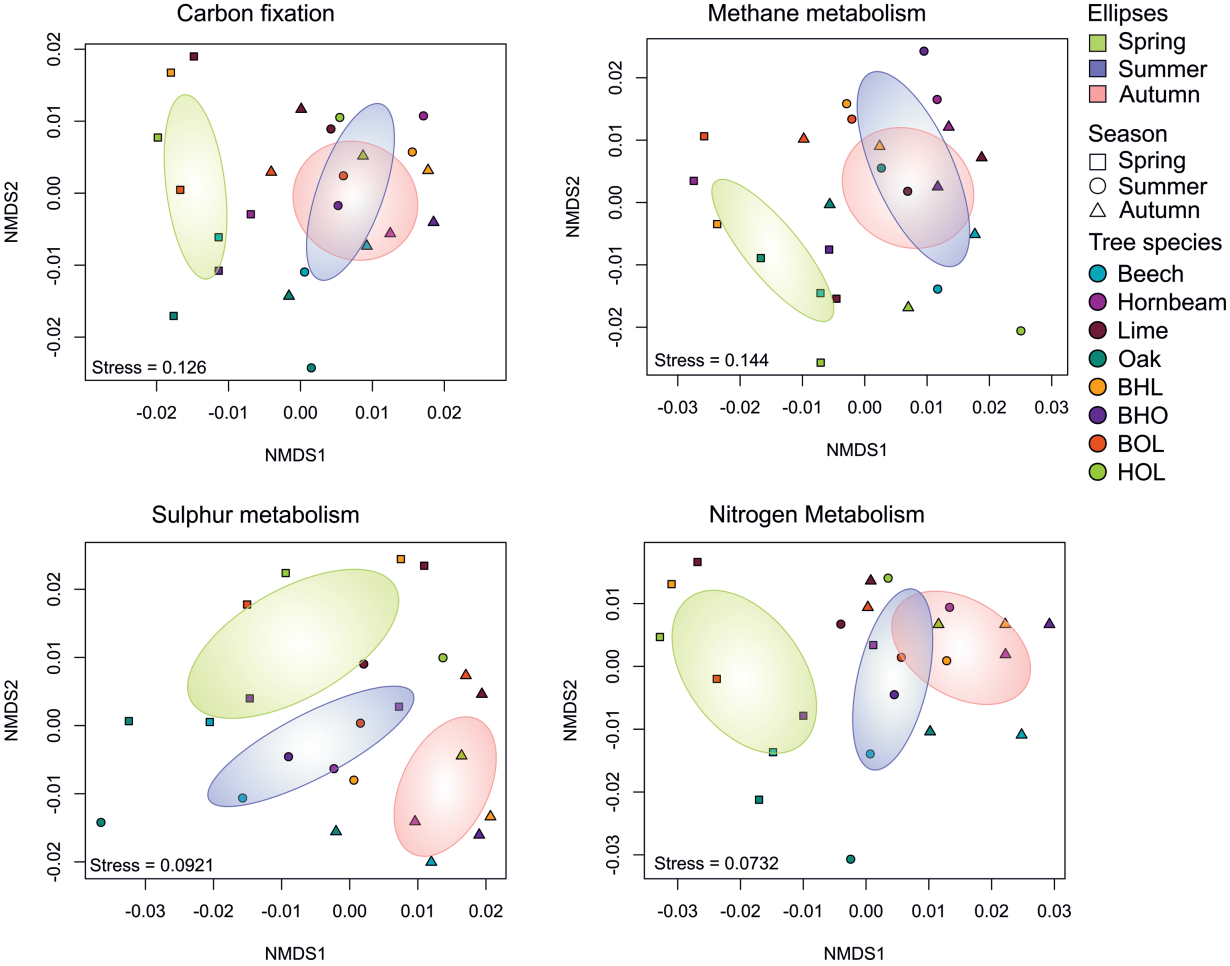
Non-metric multidimensional scaling (NMDS) of functional genes in key biogeochemical processes. Ordination is based on Bray-Curtis distance matrices of functional genes (KEGG orthologs) predicted by Tax4Fun. A subset of genes predicted for each metabolic process were used to generate distance matrices, which were subsequently summarized by tree species (*n* = 6).

In general, predicted metabolic functional profiles did not follow the stand-specific trend recorded for the bacterial community structures but showed strong grouping according to season (*p* = 0.001) (Figure 5; Supplementary Table S5). Only functional genes related to methane metabolism showed association with tree species (*p* = 0.033) (Supplementary Figure S7). This is in accordance with a report showing that methylotrophic bacteria correlated with shifts in soil pH in a beech-dominated deciduous forest (Morawe et al., 2017). In general, gene functions associated with carbon and nitrogen metabolism in spring were distinct from summer and autumn, which grouped more closely (Figure 5). As a subset of carbon metabolism, gene functions associated with methane metabolism also followed this trend. Gene function for sulfur metabolism also showed separation between seasons. As light intensity and temperature increase in spring (April), trees begin to form leaves and increase photosynthetic productivity, which peaks in summer (July) (Goodale et al., 2015). In autumn (October), light intensity and temperature decline and trees, generally, respond through increased litterfall (Goodale et al., 2015; Žifčáková et al., 2016). However, most litter decomposition takes place during the summer (Sohng et al., 2014) and may continue into autumn. Correspondingly, we concluded that belowground metabolic processes from summer extended into autumn, giving rise to similar bacterial functional profiles.

**Figure 6:**
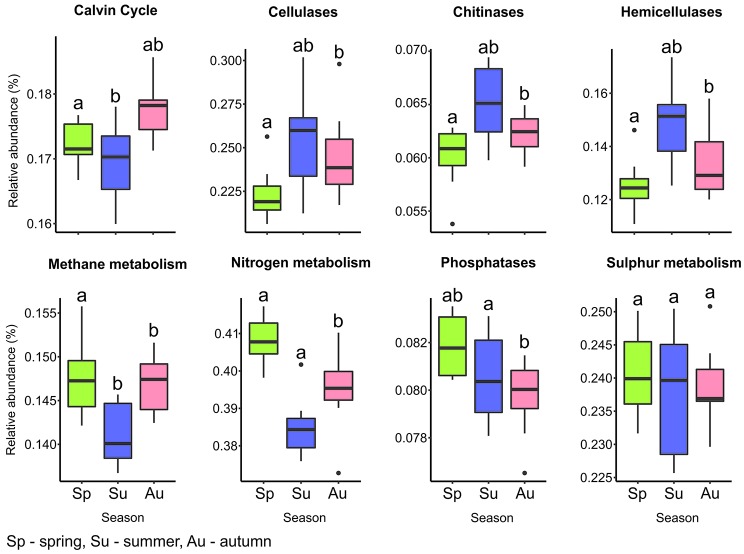
Overview of predicted bacterial functional categories involved in key energy pathways. Genes were predicted from KEGG orthologs with Tax4Fun (Aßhauer et al., 2015). Each box plot represents the mean relative abundance of predicted genes for the eight mono and mixed stands (each with six replicates) calculated for spring, summer, and autumn. Genes used are listed in Supplementary Table S4. Mean values with identical letters, determined by ANOVA with Tukey HSD *post hoc* test, share significant similarity among seasons (*p* ≤ 0.05).

We observed several predicted functional gene profiles related to carbon (including methane) and nitrogen metabolism, key pathways in microbial biogeochemical cycling (Figure 6; Supplementary Table S6). The higher abundance of genes observed for carbon metabolism agree with other studies showing that C cycling is a central part to bacterial metabolism in forest soils (Nacke et al., 2014; Siles and Margesin, 2017). Carbon turnover in soils is mediated by carbohydrate-active enzymes (CAZymes), which act on labile C compounds, recalcitrant cellulose or hemicellulose and fungal biomass (López-Mondéjar et al., 2016; Žifčáková et al., 2017). In our study, gene function related to C fixation, including ribulose 1,5-bisphosphate carboxylase/oxygenase (RuBisCo), showed significantly higher abundance in spring and autumn (*p* = 0.013). However, the relative abundances of C degradation genes (cellulases, hemicellulases, and chitinases) were higher in summer and autumn than in spring. Due to temperature dependence, CAZymes activity was demonstrated to correlate with seasonal changes, which subsequently impacts C cycling (Žifčáková et al., 2017). The quality of soil C input from root exudates and litter varies seasonally (Siles and Margesin, 2017) and explains the seasonal changes in the relative abundance of C fixation and degradation gene functions. A similar increase in C degradation gene function in autumn compared to spring was also reported in a mixed deciduous and coniferous forest ecosystem (Siles and Margesin, 2017).

The ability to utilize a wide range of C sources to obtain metabolic energy is shared among several bacterial taxa across phyla and enables several groups to inhabit the same environment with different nutrient niches (Lladó et al., 2017). Bacterial phyla comprise metabolically versatile genera that carry out the same general metabolic processes and are functionally redundant (Burke et al., 2011). Therefore, the relative abundance of genes in an environment does not always correlate with metabolic activity of the corresponding pathways, as some bacteria are able to use more efficient pathways to metabolize the same substrate (Rocca et al., 2015). As C allocation is mediated by bacteria through decomposition of organic matter, particularly through lignocellulose breakdown (Lladó et al., 2017), we also found potential cellulolytic genera in our study. These included *Burkholderia*, *Variovorax*, and *Flavobacterium* (Haichar et al., 2007; Ulrich et al., 2008; Schellenberger et al., 2010). Additional genera with potential cellulolytic potential were classified as rare (less than 1%) in our dataset, and included actinobacterial genera (*Arthrobacter*, *Cellulomonas*, *Kitasatospora*, *Oerskovia*, *Micromonospora*, and *Streptomyces*), alphabroteobacterial genera (*Mesorhizobium*, *Methylobacterium*, *Sphingomonas*), the gammaproteobacterial genus *Dyella* and genera from *Firmicutes* (*Bacillus*, *Paenibacillus*).

Tax4Fun predicted gene functions encoding methane monooxygenase (MMO), a key enzyme in the oxidation of methane to methanol, harbored by methylotrophic and methanotrophic bacteria (Supplementary Table S6; Hakemian and Rosenzweig, 2007; Hoppe et al., 2015). Methylotrophs metabolize single carbon substrates, as a by-product of lignin degradation (Hoppe et al., 2015). Potential methylotrophs were observed in *Rhizobiales* (*Methylocella*, and *Methyloferula*), gammaproteobacterial *Methylococcales* (*Methylococcaceae*), and *Verrucomicrobia* (*Methylacidiphilum*). Seasonal inputs of organic matter from decomposition explain the strong effect of season on the abundance of methanotrophs. For example, Hoppe et al. (2015) demonstrated an increase of methylotrophic *Rhizobiales* at different stages of deadwood decomposition in a beech and spruce deciduous forest.

The presence of gene functions for assimilatory (*sir, cys* genes) and dissimilatory (*dsr*) sulphate reduction revealed the potential presence of sulfur metabolizing organisms. However, the relative abundance of these genes did not exhibit significant differences across seasons (*p* = 0.818; Figure 6). This is explained by reports that sulphate reducing bacteria tend to be rare and are likely CRTs, within the environment despite carrying out the major part of sulfur metabolism (Yousuf et al., 2014b; Hausmann et al., 2016). Candidates detected included *Desulfurellales*, *Desulfurbacterales*, and *Desulfuromonadales* within *Deltaproteobacteria*, *Thiohalophilus*, and *Thioalkalispira* from *Gammaproteobacteria*, *Comamonadaceae* (*Betaproteobacteria*), *Rhodopseudomonas* (*Alphaproteobacteria*), and *Desulfosporosinus* (*Firmicutes*). Several studies have identified these taxa as major components of sulfur cycling in soil and sediment ecosystems (Baker et al., 2015; Balk et al., 2015; Ling et al., 2015; Hausmann et al., 2016).

Gene functions such as *nir*, *nif*, *hao,* and *amo* for the main processes in nitrogen cycling (nitrification, nitrate reduction, and anammox) were observed (Supplementary Table S6; Norton et al., 2002; Giles et al., 2012). Groups associated with N cycling from our study potentially belong to *Nitrospira*, all genera within *Nitrosomonadaceae* and *Rhizobiales* (*Bradyrhizobium* and *Rhizobium*), *Rhodospirillaceae* (*Azospirillum*), and *Actinobacteria* (*Arthrobacter*). Gene function for N metabolism was significantly more abundant during spring and autumn (*p* ≤ 0.01) (Figure 6), and similar to C cycling genes, corresponding to periods of increased nutrient availability. Taxonomic analysis revealed that the diversity of N cycling bacterial community members spans several taxonomic groups. This also demonstrates the complexity of bacteria-mediated N cycling, which is closely interlinked with C cycling (Yousuf et al., 2014a). Thus, bacteria have developed alternative pathways enabling facultative metabolism of both carbon and nitrogen substrates (Freedman et al., 2013; Yousuf et al., 2014a; Rocca et al., 2015). Furthermore, the shared trend in seasonal abundance observed for C and N metabolic gene functions in our study was expected, as soil C allocation affects soil pH and C/N ratio, which subsequently impact nitrification, denitrification, and C cycling (Townsend et al., 2011; Cardenas et al., 2018).

## Conclusions

Tree species exhibited a strong positive correlations with soil bacterial diversity and composition at entire and active soil bacterial community level, which supported our first hypothesis (1) that tree species identity drives bacterial community structure at entire and potentially active bacterial community level. This potentially is a result of both direct and indirect factors such as litterfall and root exudates, which change soil pH, C/N ratio, N and P availability. Beech and oak mono stands displayed low pH and high C/N ratio and correspondingly, showed a higher relative abundance of oligotrophic and lower relative abundance of copiotrophic bacterial taxa compared to lime and hornbeam mono stands. Mono stands compared to mixed stands showed a higher number of indicator OTUs corresponding to organisms closely associated with each stand. Most indicator OTUs belonged to *Rhizobiales*, indicating the widespread physiological adaptation of its members to different environments. Our second hypothesis that (2) metabolic functions are also driven by tree species was not supported. We did not observe stand-specific effects on predicted bacterial metabolic functions, except for gene functions related to methane metabolism. Interestingly, predicted functional metabolic profiles correlated significantly with season. This was attributed to functional redundancy across different taxonomic groups. We observed more gene functions associated with C fixation and degradation (including methane metabolism), compared to nitrogen metabolism. This supports evidence that forest ecosystems play a central role in carbon storage and contribute to global carbon cycling. The abundance of C and cycling genes showed similar increase in spring and autumn, which was linked to shared metabolic pathways across different bacterial taxa. An increase in gene functions for both processes corresponded to periods of increased soil nutrient availability, as a response to increased root productivity (spring) and litterfall (autumn). We demonstrated that hypothesis (3) soil physicochemical parameters are strongly influenced by tree species identity (mono stands) and richness (mixed stands), which subsequently drive the observed differences in bacterial community structure at total and active community level. Furthermore, our hypothesis (2) that soil bacterial structure across forest stands is shaped by season to a lesser extent than by tree species was not fully supported. Season showed a non-significant effect on soil bacterial community composition at total and active community level. The approach to evaluate soil bacteria at total and active community level provided a comprehensive overview of compositional and potential functional changes in soil bacterial communities. Moreover, the ability to monitor taxonomic and functional relationships in individual microbial taxa provides insights into specific impacts of trees on shaping soil microbial communities and improves our understanding of how potential conversion of forest stands effects change in soil microbial community and function.

## Author Contributions

RD designed and conceived the study. Soil sampling for prokaryotic community analysis was performed by AD. AD carried out the field and laboratory work. MS and EV set up experimental design and site and provided data. AD and DS prepared and analyzed the data. AD generated the draft version of the manuscript. All authors interpreted the results and contributed to writing the final version of the manuscript.

### Conflict of Interest Statement

The authors declare that the research was conducted in the absence of any commercial or financial relationships that could be construed as a potential conflict of interest.

## References

[ref1] Aßhauer K. P. Wemheuer B. Daniel R. Meinicke P. (2015). Tax4Fun: predicting functional profiles from metagenomic 16S rRNA data. Bioinformatics 31, 2882–2884. 10.1093/bioinformatics/btv287, PMID: 25957349 PMC4547618

[ref2] Augusto L. De Schrijver A. Vesterdal L. Smolander A. Prescott C. Ranger J. (2015). Influences of evergreen gymnosperm and deciduous angiosperm tree species on the functioning of temperate and boreal forests. Biol. Rev. 90, 444–466. 10.1111/brv.12119, PMID: 24916992

[ref3] Augusto L. Ranger J. Binkley D. Rothe A. (2002). Impact of several common tree species of European temperate forests on soil fertility. Ann. For. Sci. 59, 233–253. 10.1051/forest:2002020

[ref4] Baker B. J. Lazar C. S. Teske A. P. Dick G. J. (2015). Genomic resolution of linkages in carbon, nitrogen, and sulfur cycling among widespread estuary sediment bacteria. Microbiome 3:14. 10.1186/s40168-015-0077-6 25922666 PMC4411801

[ref5] Baldrian P. Kolaiřík M. Štursová M. Kopecký J. Valášková V. Větrovský T.. (2012). Active and total microbial communities in forest soil are largely different and highly stratified during decomposition. ISME J. 6, 248–258. 10.1038/ismej.2011.95, PMID: 21776033 PMC3260513

[ref6] Balk M. Keuskamp J. A. Laanbroek H. J. (2015). Potential activity, size, and structure of sulfate-reducing microbial communities in an exposed, grazed and a sheltered, non-grazed mangrove stand at the Red Sea coast. Front. Microbiol. 6:1478. 10.3389/fmicb.2015.01478, PMID: 26733999 PMC4686736

[ref7] Barabote R. D. Xie G. Leu D. H. Normand P. Necsulea A. Daubin V.. (2009). Complete genome of the cellulolytic thermophile acidothermus cellulolyticus 11B provides insights into its ecophysiological and evolutionary adaptations. Genome Res. 19, 1033–1043. 10.1101/gr.084848.108, PMID: 19270083 PMC2694482

[ref8] Barka E. A. Vatsa P. Sanchez L. Gaveau-Vaillant N. Jacquard C. Klenk H.-P. (2016). Taxonomy, physiology, and natural products of Actinobacteria. Microbiol. Mol. Biol. Rev. 80, 1–43. 10.1128/MMBR.00019-15 26609051 PMC4711186

[ref9] Berger T. W. Berger P. (2014). Does mixing of beech (*Fagus sylvatica*) and spruce (*Picea abies*) litter hasten decomposition? Plant Soil 377, 217–234. 10.1007/s11104-013-2001-9, PMID: 24744450 PMC3987168

[ref10] Bergkemper F. Welzl G. Lang F. Krüger J. Schloter M. Schulz S. (2016). The importance of C, N and P as driver for bacterial community structure in German beech dominated forest soils. J. Plant Nutr. Soil Sci. 179, 472–480. 10.1002/jpln.201600077

[ref11] Blazewicz S. J. Barnard R. L. Daly R. A. Firestone M. K. (2013). Evaluating rRNA as an indicator of microbial activity in environmental communities: limitations and uses. ISME J. 7, 2061–2068. 10.1038/ismej.2013.102, PMID: 23823491 PMC3806256

[ref12] Bonan G. B. (2008). Forests and climate change: forcings, feedbacks, and the climate benefits of fiorests. Science 320, 1444–1449. 10.1126/science.1155121, PMID: 18556546

[ref13] Bonito G. Reynolds H. Robeson M. S. Nelson J. Hodkinson B. P. Tuskan G. (2014). Plant host and soil origin influence fungal and bacterial assemblages in the roots of woody plants. Mol. Ecol. 23, 3356–3370. 10.1111/mec.12821 24894495

[ref14] Bragg L. Stone G. Imelfort M. Hugenholtz P. Tyson G. W. (2012). Fast, accurate error-correction of amplicon pyrosequences using Acacia. Nat. Methods 9, 425–426. 10.1038/nmeth.1990, PMID: 22543370

[ref15] Brunet J. Falkengren-Grerup U. Tyler G. (1997). Pattern and dynamics of the ground vegetation in south Swedish Carpinus betulus forests: importance of soil chemistry and management. Ecography 20, 513–520. 10.1111/j.1600-0587.1997.tb00420.x

[ref16] Burke C. Steinberg P. Rusch D. B. Kjelleberg S. Thomas T. (2011). Bacterial community assembly based on functional genes rather than species. Proc. Natl. Acad. Sci. USA 108, 14288–14293. 10.1073/pnas.1101591108 21825123 PMC3161577

[ref17] Canarini A. Kaiser C. Merchant A. Richter A. Wanek W. (2019). Root exudation of primary metabolites: mechanisms and their roles in plant responses to environmental stimuli. Front. Plant Sci. 10:157. 10.3389/fpls.2019.00157 30881364 PMC6407669

[ref18] Caporaso J. G. Kuczynski J. Stombaugh J. Bittinger K. Bushman F. D. Costello E. K.. (2010). QIIME allows analysis of high- throughput community sequencing data. Nat. Methods 7, 335–336. 10.1038/nmeth.f.303, PMID: 20383131 PMC3156573

[ref19] Cardenas E. Kranabetter J. M. Hope G. Maas K. R. Hallam S. Mohn W. W. (2015). Forest harvesting reduces the soil metagenomic potential for biomass decomposition. ISME J. 9, 2465–2476. 10.1038/ismej.2015.57, PMID: 25909978 PMC4611510

[ref20] Cardenas E. Orellana L. H. Konstantinidis K. T. Mohn W. W. (2018). Effects of timber harvesting on the genetic potential for carbon and nitrogen cycling in five North American forest ecozones. Sci. Rep. 8, 1–13. 10.1038/s41598-018-21197-0 29453368 PMC5816661

[ref21] Carvalho F. M. Souza R. C. Barcellos F. G. Hungria M. Vasconcelos A. T. R. (2010). Genomic and evolutionary comparisons of diazotrophic and pathogenic bacteria of the order Rhizobiales. BMC Microbiol. 10:37. 10.1186/1471-2180-10-37, PMID: 20144182 PMC2907836

[ref22] Cherobaeva A. S. Kizilova A. K. Stepanov A. L. Kravchenko I. K. (2011). Molecular analysis of the diversity of nitrifying bacteria in the soils of the forest and steppe zones of European Russia. Microbiology 80, 395–402. 10.1134/S0026261711030064 21861377

[ref23] Chodak M. Klimek B. Niklińska M. (2016). Composition and activity of soil microbial communities in different types of temperate forests. Biol. Fertil. Soils 52, 1093–1104. 10.1007/s00374-016-1144-2

[ref24] Cline M. S. Smoot M. Cerami E. Kuchinsky A. Landys N. Workman C.. (2007). Integration of biological networks and gene expression data using cytoscape. Nat. Protoc. 2, 2366–2382. 10.1038/nprot.2007.324, PMID: 17947979 PMC3685583

[ref25] Clivot H. Pagnout C. Aran D. Devin S. Bauda P. Poupin P. (2012). Changes in soil bacterial communities following liming of acidified forests. Appl. Soil Ecol. 59, 116–123. 10.1016/j.apsoil.2011.09.010

[ref26] Colin Y. Nicolitch O. Van Nostrand J. D. Zhou J. Z. Turpault M. P. Uroz S. (2017). Taxonomic and functional shifts in the beech rhizosphere microbiome across a natural soil toposequence. Sci. Rep. 7:9604. 10.1038/s41598-017-07639-1 28851878 PMC5574896

[ref27] Creamer R. E. E. Hannula S. E. E. Leeuwen J. P. V. P. V. Stone D. Rutgers M. Schmelz R. M. M. (2016). Ecological network analysis reveals the inter-connection between soil biodiversity and ecosystem function as affected by land use across Europe. Appl. Soil Ecol. 97, 112–124. 10.1016/j.apsoil.2015.08.006

[ref28] Curd E. E. Martiny J. B. H. Li H. Smith T. B. (2018). Bacterial diversity is positively correlated with soil heterogeneity. Ecosphere 9:e02079. 10.1002/ecs2.2079

[ref29] Dawid W. (2000). Biology and global distribution of myxobacteria in soils. FEMS Microbiol. Rev. 24, 403–427. 10.1111/j.1574-6976.2000.tb00548.x, PMID: 10978544

[ref30] De Cáceres M. (2013). How to use the indicspecies package (ver. 1.7.1). R Proj., 1–29. Available at: https://cran.r-project.org/web/packages/indicspecies/index.html (last accessed on 25.06.2019).

[ref31] De Vos B. Cools N. Ilvesniemi H. Vesterdal L. Vanguelova E. Carnicelli S. (2015). Benchmark values for forest soil carbon stocks in Europe: results from a large scale forest soil survey. Geoderma 251–252, 33–46. 10.1016/j.geoderma.2015.03.008

[ref32] DeAngelis K. M. Pold G. Topçuoğlu B. D. van Diepen L. T. A. Varney R. M. Blanchard J. L.. (2015). Long-term forest soil warming alters microbial communities in temperate forest soils. Front. Microbiol. 6:104. 10.3389/fmicb.2015.00104, PMID: 25762989 PMC4327730

[ref33] Deshmukh Y. Sao S. (2015). Degradation of lignin through carbon utilization by the microbial ligninolytic enzymes for environmental management. IOSR J. Environ. Sci. Toxicol. Food Technol. 5, 27–31.

[ref34] Dilly O. Bach H. J. Buscot F. Eschenbach C. Kutsch W. L. Middelhoff U. (2000). Characteristics and energetic strategies of the rhizosphere in ecosystems of the Bornhoved lake district. Appl. Soil Ecol. 15, 201–210. 10.1016/S0929-1393(00)00096-2

[ref35] Edgar R. C. Haas B. J. Clemente J. C. Quince C. Knight R. (2011). UCHIME improves sensitivity and speed of chimera detection. Bioinformatics 27, 2194–2200. 10.1093/bioinformatics/btr381, PMID: 21700674 PMC3150044

[ref36] Falkengren-Grerup U. Brunet J. Diekmann M. (1998). Nitrogen mineralisation in deciduous forest soils in south Sweden in gradients of soil acidity and deposition. Environ. Pollut. 102, 415–420. 10.1016/S0269-7491(98)80062-6

[ref37] Felske A. Wolterink A. Van Lis R. Antoon D. Akkermans L. (1998). Phylogeny of the main bacterial 16S rRNA sequences in Drentse A grassland soils (The Netherlands). Appl. Environ. Microbiol. 64, 871–879. PMID: . 9501427 10.1128/aem.64.3.871-879.1998 PMC106340

[ref38] Ferrenberg S. O’neill S. P. Knelman J. E. Todd B. Duggan S. Bradley D.. (2013). Changes in assembly processes in soil bacterial communities following a wildfire disturbance. ISME J. 7, 1102–1111. 10.1038/ismej.2013.11, PMID: 23407312 PMC3660671

[ref39] Fierer N. Leff J. W. Adams B. J. Nielsen U. N. Bates S. T. Lauber C. L. (2012). Cross-biome metagenomic analyses of soil microbial communities and their functional attributes. Proc. Natl. Acad. Sci. USA 109, 21390–21395. 10.1073/pnas.1215210110 23236140 PMC3535587

[ref40] Flores G. E. Hunter R. C. Liu Y. Mets A. Schouten S. Reysenbach A. L. (2012). Hippea jasoniae sp. nov. and Hippea alviniae sp. nov., thermoacidophilic members of the class Deltaproteobacteria isolated from deep-sea hydrothermal vent deposits. Int. J. Syst. Evol. Microbiol. 62, 1252–1258. 10.1099/ijs.0.033001-0 21764980

[ref41] Freedman Z. Eisenlord S. D. Zak D. R. Xue K. He Z. Zhou J. (2013). Towards a molecular understanding of N cycling in northern hardwood forests under future rates of N deposition. Soil Biol. Biochem. 66, 130–138. 10.1016/j.soilbio.2013.07.010

[ref42] Fudou R. Iizuka T. Sato S. Ando T. Shimba N. Yamanaka S. (2001). Haliangicin, a novel antifungal metabolite produced by a marine myxobacterium. 2. Isolation and structural elucidation. J. Antibiot. 54, 153–156. 10.7164/antibiotics.54.153, PMID: 11302488

[ref43] Fudou R. Jojima Y. Iizuka T. Yamanaka S. (2002). Haliangium ochraceum gen. nov., sp. nov. and *Haliangium tepidum* sp. nov.: novel moderately halophilic myxobacteria isolated from coastal saline environments. J. Gen. Appl. Microbiol. 48, 109–116. 10.2323/jgam.48.109, PMID: 12469307

[ref44] Gergs R. Rothhaupt K.-O. (2015). Invasive species as driving factors for the structure of benthic communities in Lake Constance, Germany. Hydrobiologia 746, 245–254. 10.1007/s10750-014-1931-4

[ref45] Giles M. Morley N. Baggs E. M. Daniell T. J. (2012). Soil nitrate reducing processes – drivers, mechanisms for spatial variation, and significance for nitrous oxide production. Front. Microbiol. 3:407. 10.3389/fmicb.2012.00407, PMID: 23264770 PMC3524552

[ref46] Gill A. S. Lee A. McGuire K. L. (2017). Phylogenetic and functional diversity of total (DNA) and expressed (RNA) bacterial communities in urban green infrastructure bioswale soils. Appl. Environ. Microbiol. 83, e00287–e00217. 10.1128/AEM.00287-17 28576763 PMC5541207

[ref47] Goodale C. L. Fredriksen G. Weiss M. S. McCalley C. K. Sparks J. P. Thomas S. A. (2015). Soil processes drive seasonal variation in retention of in a deciduous forest catchment N tracers. Ecology 96, 2653–2668. 10.1890/14-1852.1, PMID: 26649387

[ref48] Gorka S. Dietrich M. Mayerhofer W. Gabriel R. Wiesenbauer J. Martin V. (2019). Rapid transfer of plant photosynthates to soil bacteria *via* ectomycorrhizal hyphae and its interaction with nitrogen availability. Front. Microbiol. 10:168. 10.3389/fmicb.2019.00168 30863368 PMC6399413

[ref49] Green S. J. Prakash O. Jasrotia P. Overholt W. A. Cardenas E. Hubbard D.. (2012). Denitrifying bacteria from the genus Rhodanobacter dominate bacterial communities in the highly contaminated subsurface of a nuclear legacy waste site. Appl. Environ. Microbiol. 78, 1039–1047. 10.1128/AEM.06435-11, PMID: 22179233 PMC3273022

[ref50] Haichar F. E. Z. Achouak W. Christen R. Heulin T. Marol C. Marais M.-F.. (2007). Identification of cellulolytic bacteria in soil by stable isotope probing. Environ. Microbiol. 9, 625–634. 10.1111/j.1462-2920.2006.01182.x, PMID: 17298363

[ref51] Hakemian A. S. Rosenzweig A. C. (2007). The biochemistry of methane oxidation. Annu. Rev. Biochem. 76, 223–241. 10.1146/annurev.biochem.76.061505.175355, PMID: 17328677

[ref52] Hartmann M. Frey B. Mayer J. Mäder P. Widmer F. (2015). Distinct soil microbial diversity under long-term organic and conventional farming. ISME J. 9, 1177–1194. 10.1038/ismej.2014.210, PMID: 25350160 PMC4409162

[ref53] Hausmann B. Knorr K. H. Schreck K. Tringe S. G. Glavina del Rio T. Loy A.. (2016). Consortia of low-abundance bacteria drive sulfate reduction-dependent degradation of fermentation products in peat soil microcosms. ISME J. 10, 2365–2375. 10.1038/ismej.2016.42, PMID: 27015005 PMC4930147

[ref54] Herzog S. Wemheuer F. Wemheuer B. Daniel R. (2015). Effects of fertilization and sampling time on composition and diversity of entire and active bacterial communities in German grassland soils. PLoS One 10:e0145575. 10.1371/journal.pone.0145575, PMID: 26694644 PMC4687936

[ref55] Hoppe B. Krüger D. Kahl T. Arnstadt T. Buscot F. Bauhus J. (2015). A pyrosequencing insight into sprawling bacterial diversity and community dynamics in decaying deadwood logs of *Fagus sylvatica* and *Picea abies*. Sci. Rep. 5, 23–25. 10.1038/srep09456 PMC4389208 25851097

[ref56] Huber K. J. Geppert A. M. Wanner G. Fösel B. U. Wüst P. K. Overmann J. (2016). The first representative of the globally widespread subdivision 6 Acidobacteria, Vicinamibacter silvestris gen. nov., sp. nov., isolated from subtropical savannah soil. Int. J. Syst. Evol. Microbiol. 66, 2971–2979. 10.1099/ijsem.0.001131 27150379

[ref57] Jacob A. Hertel D. Leuschner C. (2013). On the significance of belowground overyielding in temperate mixed forests: separating species identity and species diversity effects. Oikos 122, 463–473. 10.1111/j.1600-0706.2012.20476.x

[ref58] Jacob M. Weland N. Platner C. Schaefer M. Leuschner C. Thomas F. M. (2009). Nutrient release from decomposing leaf litter of temperate deciduous forest trees along a gradient of increasing tree species diversity. Soil Biol. Biochem. 41, 2122–2130. 10.1016/j.soilbio.2009.07.024

[ref59] Jeanbille M. Buée M. Bach C. Cébron A. Frey-Klett P. Turpault M. P.. (2016). Soil parameters drive the structure, diversity and metabolic potentials of the bacterial communities across temperate beech forest soil sequences. Microb. Ecol. 71, 482–493. 10.1007/s00248-015-0669-5, PMID: 26370112

[ref60] Kaiser C. Kilburn M. R. Clode P. L. Fuchslueger L. Koranda M. Cliff J. B.. (2015). Exploring the transfer of recent plant photosynthates to soil microbes: mycorrhizal pathway vs direct root exudation. New Phytol. 205, 1537–1551. 10.1111/nph.13138, PMID: 25382456 PMC4357392

[ref61] Kaiser K. Wemheuer B. Korolkow V. Wemheuer F. Nacke H. Schöning I. (2016). Driving forces of soil bacterial community structure, diversity, and function in temperate grasslands and forests. Sci. Rep. 6:33696. 10.1038/srep33696 27650273 PMC5030646

[ref62] Khlifa R. Paquette A. Messier C. Reich P. B. Munson A. D. (2017). Do temperate tree species diversity and identity influence soil microbial community function and composition? Ecol. Evol. 7, 7965–7974. 10.1002/ece3.3313, PMID: 29043048 PMC5632628

[ref63] Kielak A. M. Barreto C. C. Kowalchuk G. A. van Veen J. A. Kuramae E. E. (2016a). The ecology of Acidobacteria: moving beyond genes and genomes. Front. Microbiol. 7:744. 10.3389/fmicb.2016.00744 27303369 PMC4885859

[ref64] Kielak A. M. Scheublin T. R. Mendes L. W. van Veen J. A. Kuramae E. E. (2016b). Bacterial community succession in pine-wood decomposition. Front. Microbiol. 7:231. 10.3389/fmicb.2016.00231 26973611 PMC4771932

[ref65] Kim S. J. Ahn J. H. Lee T. H. Weon H. Y. Hong S. B. Seok S. J. (2013). Reyranella soli sp. nov., isolated from forest soil, and emended description of the genus Reyranella Pagnier et al. 2011. Int. J. Syst. Evol. Microbiol. 63, 3164–3167. 10.1099/ijs.0.045922-0 23435248

[ref66] Kim J. S. Lee K. C. Kim D. S. Ko S. H. Jung M. Y. Rhee S. K.. (2015). Pyrosequencing analysis of a bacterial community associated with lava-formed soil from the Gotjawal forest in Jeju, Korea. Microbiology 4, 301–312. 10.1002/mbo3.238, PMID: 25604185 PMC4398510

[ref67] Klimek B. Chodak M. Jaźwa M. Solak A. Tarasek A. Niklińska M. (2016). The relationship between soil bacteria substrate utilisation patterns and the vegetation structure in temperate forests. Eur. J. For. Res. 135, 179–189. 10.1007/s10342-015-0929-4

[ref68] Kolton M. Sela N. Elad Y. Cytryn E. (2013). Comparative genomic analysis indicates that niche adaptation of terrestrial Flavobacteria is strongly linked to plant glycan metabolism. PLoS One 8:e76704. 10.1371/journal.pone.0076704, PMID: 24086761 PMC3784431

[ref69] Koyama A. Wallenstein M. D. Simpson R. T. Moore J. C. (2014). Soil bacterial community composition altered by increased nutrient availability in Arctic tundra soils. Front. Microbiol. 5:516. 10.3389/fmicb.2014.00516, PMID: 25324836 PMC4183186

[ref70] Kubisch P. Hertel D. Leuschner C. (2015). Do ectomycorrhizal and arbuscular mycorrhizal temperate tree species systematically differ in root order-related fine root morphology and biomass? Front. Plant Sci. 6:64. 10.3389/fpls.2015.00064, PMID: 25717334 PMC4324066

[ref71] Landesman W. J. Nelson D. M. Fitzpatrick M. C. (2014). Soil properties and tree species drive ß-diversity of soil bacterial communities. Soil Biol. Biochem. 76, 201–209. 10.1016/j.soilbio.2014.05.025

[ref72] Lang F. Bauhus J. Frossard E. George E. Kaiser K. Kaupenjohann M. (2016). Phosphorus in forest ecosystems: new insights from an ecosystem nutrition perspective. J. Plant Nutr. Soil Sci. 2015, 129–135. 10.1002/jpln.201500541

[ref73] Langenbruch C. Helfrich M. Flessa H. (2012). Effects of beech (*Fagus sylvatica*), ash (*Fraxinus excelsior*) and lime (Tilia spec.) on soil chemical properties in a mixed deciduous forest. Plant Soil 352, 389–403. 10.1007/s11104-011-1004-7

[ref74] Lauber C. L. Strickland M. S. Bradford M. A. Fierer N. (2008). The influence of soil properties on the structure of bacterial and fungal communities across land-use types. Soil Biol. Biochem. 40, 2407–2415. 10.1016/j.soilbio.2008.05.021

[ref75] Li S. G. Zhao L. Han K. Li P. F. Li Z. F. Hu W.. (2014). Diversity of epothilone producers among Sorangium strains in producer-positive soil habitats. Microb. Biotechnol. 7, 130–141. 10.1111/1751-7915.12103, PMID: 24308800 PMC3937717

[ref76] Liese R. Lübbe T. Albers N. W. Meier I. C. (2018). The mycorrhizal type governs root exudation and nitrogen uptake of temperate tree species. Tree Physiol. 38, 83–95. 10.1093/treephys/tpx131, PMID: 29126247

[ref77] Ling Y. C. Bush R. Grice K. Tulipani S. Berwick L. Moreau J. W. (2015). Distribution of iron- and sulfate-reducing bacteria across a coastal acid sulfate soil (CASS) environment: implications for passive bioremediation by tidal inundation. Front. Microbiol. 6:624. 10.3389/fmicb.2015.00624, PMID: 26191042 PMC4490247

[ref78] Liu Z. Lozupone C. Hamady M. Bushman F. D. Knight R. (2007). Short pyrosequencing reads suffice for accurate microbial community analysis. Nucleic Acids Res. 35:e120. 10.1093/nar/gkm541, PMID: 17881377 PMC2094085

[ref79] Lladó S. López-Mondéjar R. Baldrian P. (2017). Forest soil bacteria: diversity, involvement in ecosystem processes, and response to global change. Microbiol. Mol. Biol. Rev. 81, e00063–e00016. 10.1128/MMBR.00063-16 28404790 PMC5485800

[ref80] Lladó S. López-Mondéjar R. Baldrian P. (2018). Drivers of microbial community structure in forest soils. Appl. Microbiol. Biotechnol. 102, 4331–4338. 10.1007/s00253-018-8950-4, PMID: 29600493

[ref81] Lladó S. Žifčáková L. Větrovský T. Eichlerová I. Baldrian P. (2016). Functional screening of abundant bacteria from acidic forest soil indicates the metabolic potential of acidobacteria subdivision 1 for polysaccharide decomposition. Biol. Fertil. Soils 52, 251–260. 10.1007/s00374-015-1072-6

[ref82] López-Mondéjar R. Zühlke D. Becher D. Riedel K. Baldrian P. (2016). Cellulose and hemicellulose decomposition by forest soil bacteria proceeds by the action of structurally variable enzymatic systems. Sci. Rep. 6:25279. 10.1038/srep25279 27125755 PMC4850484

[ref83] Lozupone C. Lladser M. E. Knights D. Stombaugh J. Knight R. (2011). UniFrac: an effective distance metric for microbial community comparison. ISME J. 5, 169–172. 10.1038/ismej.2010.133, PMID: 20827291 PMC3105689

[ref85] Mamlouk D. Gullo M. (2013). Acetic acid bacteria: physiology and carbon sources oxidation. Indian J. Microbiol. 53, 377–384. 10.1007/s12088-013-0414-z, PMID: 24426139 PMC3779290

[ref86] Martin M. (2011). Cutadapt removes adapter sequences from high-throughput sequencing reads. EMBnet.journal 17, 10–12. 10.14806/ej.17.1.200

[ref87] Meier I. C. Avis P. G. Phillips R. P. (2013). Fungal communities influence root exudation rates in pine seedlings. FEMS Microbiol. Ecol. 83, 585–595. 10.1111/1574-6941.12016, PMID: 23013386

[ref88] Miyashita N. T. (2015). Contrasting soil bacterial community structure between the phyla Acidobacteria and Proteobacteria in tropical Southeast Asian and temperate Japanese forests. Genes Genet. Syst. 90, 61–77. 10.1266/ggs.90.61, PMID: 26399766

[ref89] Mohagheghi A. Grohmann K. Himmel M. Leighton L. Updegraff D. M. (1986). Isolation and characterization of *Acidothermus cellulolyticus* gen. nov., sp. nov., a new genus of thermophilic, acidophilic, cellulolytic bacteria. Int. J. Syst. Bacteriol. 36, 435–443. 10.1099/00207713-36-3-435

[ref90] Mölder A. Bernhardt-Römermann M. Schmidt W. (2006). Forest ecosystem research in Hainich National Park (Thuringia) first results on flora and vegetation in stands with contrsting tree species diversity. Waldökologie 3, 83–99.

[ref91] Moran M. A. Satinsky B. Gifford S. M. Luo H. Rivers A. Chan L. K.. (2013). Sizing up metatranscriptomics. ISME J. 7, 237–243. 10.1038/ismej.2012.94, PMID: 22931831 PMC3554401

[ref92] Morawe M. Hoeke H. Wissenbach D. K. Lentendu G. Wubet T. Kröber E. (2017). Acidotolerant bacteria and fungi as a sink of methanol-derived carbon in a deciduous forest soil. Front. Microbiol. 8:1361. 10.3389/fmicb.2017.01361 28790984 PMC5523551

[ref93] Nacke H. Fischer C. Thürmer A. Meinicke P. Daniel R. (2014). Land use type significantly affects microbial gene transcription in soil. Microb. Ecol. 67, 919–930. 10.1007/s00248-014-0377-6, PMID: 24553913

[ref94] Nacke H. Goldmann K. Schöning I. Pfeiffer B. Kaiser K. Villamizar G. A. C.. (2016). Fine spatial scale variation of soil microbial communities under European beech and Norway spruce. Front. Microbiol. 7:2067. 10.3389/fmicb.2016.02067, PMID: 28066384 PMC5177625

[ref95] Nacke H. Thürmer A. Wollherr A. Will C. Hodac L. Herold N.. (2011). Pyrosequencing-based assessment of bacterial community structure along different management types in German forest and grassland soils. PLoS One 6:e17000. 10.1371/journal.pone.0017000, PMID: 21359220 PMC3040199

[ref96] Naether A. Foesel B. U. Naegele V. Wüst P. K. Weinert J. Bonkowski M.. (2012). Environmental factors affect acidobacterial communities below the subgroup level in grassland and forest soils. Appl. Environ. Microbiol. 78, 7398–7406. 10.1128/AEM.01325-12, PMID: 22885760 PMC3457104

[ref97] Nemergut D. Shade A. Violle C. (2014). When, where and how does microbial community composition matter? Front. Microbiol. 5:497. 10.3389/fmicb.2014.00497, PMID: 25309525 PMC4176461

[ref98] Norton J. M. Alzerreca J. J. Suwa Y. Klotz M. G. (2002). Diversity of ammonia monooxygenase operon in autotrophic ammonia-oxidizing bacteria. Arch. Microbiol. 177, 139–149. 10.1007/s00203-001-0369-z, PMID: 11807563

[ref99] Oh Y. M. Kim M. Lee-Cruz L. Lai-Hoe A. Go R. Ainuddin N.. (2012). Distinctive bacterial communities in the rhizoplane of four tropical tree species. Microb. Ecol. 64, 1018–1027. 10.1007/s00248-012-0082-2, PMID: 22767122

[ref100] Oksanen J. Guillaume Blanchet F. Friendly F. Kindt R. Legendre P. McGlinn D. (2019). vegan: community ecology package. R Package Version 2.5-4. Available at: https://cran.r-project.org/web/packages/vegan/index.html (last accessed on 25.06.2019).

[ref101] Pfeiffer B. Fender A. C. Lasota S. Hertel D. Jungkunst H. F. Daniel R. (2013). Leaf litter is the main driver for changes in bacterial community structures in the rhizosphere of ash and beech. Appl. Soil Ecol. 72, 150–160. 10.1016/j.apsoil.2013.06.008

[ref102] Pochon X. Zaiko A. Fletcher L. M. Laroche O. Wood S. A. (2017). Wanted dead or alive? Using metabarcoding of environmental DNA and RNA to distinguish living assemblages for biosecurity applications. PLoS One 12:187636. 10.1371/journal.pone.0187636 PMC5667844 29095959

[ref103] Prakash O. Green S. J. Jasrotia P. Overholt W. A. Canion A. Watson D. B.. (2012). Rhodanobacter denitrificans sp. nov., isolated from nitrate-rich zones of a contaminated aquifer. Int. J. Syst. Evol. Microbiol. 62, 2457–2462. 10.1099/ijs.0.035840-0, PMID: 22140175

[ref104] Quast C. Pruesse E. Yilmaz P. Gerken J. Schweer T. Yarza P. (2013). The SILVA ribosomal RNA gene database project: improved data processing and web-based tools. Nucleic Acids Res. 41, 590–596. 10.1093/nar/gks1219 PMC3531112 23193283

[ref105] Rasche F. Knapp D. Kaiser C. Koranda M. Kitzler B. Zechmeister-Boltenstern S.. (2011). Seasonality and resource availability control bacterial and archaeal communities in soils of a temperate beech forest. ISME J. 5, 389–402. 10.1038/ismej.2010.138, PMID: 20882059 PMC3105729

[ref600] R Development Core Team (2017). R: A Language and Environment for Statistical Computing, ed. R Foundation for Statistical Computing. (Vienna: R Foundation for Statistical Computing).

[ref106] Rime T. Hartmann M. Frey B. (2016). Potential sources of microbial colonizers in an initial soil ecosystem after retreat of an alpine glacier. ISME J. 10, 1625–1641. 10.1038/ismej.2015.238, PMID: 26771926 PMC4918445

[ref107] Rocca J. D. Hall E. K. Lennon J. T. Evans S. E. Waldrop M. P. Cotner J. B.. (2015). Relationships between protein-encoding gene abundance and corresponding process are commonly assumed yet rarely observed. ISME J. 9, 1693–1699. 10.1038/ismej.2014.252, PMID: 25535936 PMC4511926

[ref108] Romanowicz K. J. Freedman Z. B. Upchurch R. A. Argiroff W. A. Zak D. R. (2016). Active microorganisms in forest soils differ from the total community yet are shaped by the same environmental factors: the influence of pH and soil moisture. FEMS Microbiol. Ecol. 92, 1–9. 10.1093/femsec/fiw149 27387909

[ref109] Sait M. Davis K. E. R. Janssen P. H. (2006). Effect of pH on isolation and distribution of members of subdivision 1 of the phylum Acidobacteria occurring in soil. Appl. Environ. Microbiol. 72, 1852–1857. 10.1128/AEM.72.3.1852-1857.2006, PMID: 16517631 PMC1393200

[ref110] Salehi A. Amiri G. Burslem D. Swaine M. (2007). Relationships between tree species competition, soil properties and topographic factors in a temperate deciduous forest in northern Iran. Asian J. Plant Sci. 6, 455–462.

[ref111] Scheibe A. Steffens C. Seven J. Jacob A. Hertel D. Leuschner C. (2015). Effects of tree identity dominate over tree diversity on the soil microbial community structure. Soil Biol. Biochem. 81, 219–227. 10.1016/j.soilbio.2014.11.020

[ref112] Schellenberger S. Kolb S. Drake H. L. (2010). Metabolic responses of novel cellulolytic and saccharolytic agricultural soil Bacteria to oxygen. Environ. Microbiol. 12, 845–861. 10.1111/j.1462-2920.2009.02128.x, PMID: 20050868

[ref113] Schmidt M. Veldkamp E. Corre M. D. (2015). Tree species diversity effects on productivity, soil nutrient availability and nutrient response efficiency in a temperate deciduous forest. For. Ecol. Manag. 338, 114–123. 10.1016/j.foreco.2014.11.021

[ref114] Schmidt M. Veldkamp E. Corre M. D. (2016). Tree-microbial biomass competition for nutrients in a temperate deciduous forest, central Germany. Plant Soil 408, 227–242. 10.1007/s11104-016-2923-0

[ref115] Schneider D. Engelhaupt M. Allen K. Kurniawan S. Krashevska V. Heinemann M.. (2015). Impact of lowland rainforest transformation on diversity and composition of soil prokaryotic communities in Sumatra (Indonesia). Front. Microbiol. 6:1339. 10.3389/fmicb.2015.01339, PMID: 26696965 PMC4672069

[ref116] Shade A. Jones S. E. Caporaso G. J. Handelsman J. Knight R. Fierer N. (2014). Conditionally rare taxa disproportionately contribute to temporal changes in microbial diversity. MBio 5, e1371–e1314. 10.1128/mBio.01371-14 PMC4161262 25028427

[ref117] Shange R. S. Ankumah R. O. Ibekwe A. M. Zabawa R. Dowd S. E. (2012). Distinct soil bacterial communities revealed under a diversely managed agroecosystem. PLoS One 7:e40338. 10.1371/journal.pone.0040338, PMID: 22844402 PMC3402512

[ref118] Shannon P. Markiel A. Ozier O. Baliga N. S. Wang J. T. Ramage D.. (2003). Cytoscape: a software environment for integrated models of biomolecular interaction networks. Genome Res. 13, 2498–2504. 10.1101/gr.1239303, PMID: 14597658 PMC403769

[ref119] Shen Z. Wang D. Ruan Y. Xue C. Zhang J. Li R.. (2014). Deep 16S rRNA pyrosequencing reveals a bacterial community associated with banana Fusarium wilt disease suppression induced by bio-organic fertilizer application. PLoS One 9:e98420. 10.1371/journal.pone.0098420, PMID: 24871319 PMC4037203

[ref120] Shi S. Richardson A. E. O’Callaghan M. DeAngelis K. M. Jones E. E. Stewart A.. (2011). Effects of selected root exudate components on soil bacterial communities. FEMS Microbiol. Ecol. 77, 600–610. 10.1111/j.1574-6941.2011.01150.x, PMID: 21658090

[ref121] Siles J. A. Margesin R. (2017). Seasonal soil microbial responses are limited to changes in functionality at two Alpine forest sites differing in altitude and vegetation. Sci. Rep. 7:2204. 10.1038/s41598-017-02363-2 28526872 PMC5438347

[ref122] Šnajdr J. Dobiášová P. Urbanová M. Petránková M. Cajthaml T. Frouz J. (2013). Dominant trees affect microbial community composition and activity in post-mining afforested soils. Soil Biol. Biochem. 56, 105–115. 10.1016/j.soilbio.2012.05.004

[ref123] Sohng J. Han A. R. Jeong M. A. Park Y. Park B. B. Park P. S. (2014). Seasonal pattern of decomposition and N, P, and C dynamics in leaf litter in a mongolian oak forest and a Korean pine plantation. Forests 5, 2561–2580. 10.3390/f5102561

[ref124] Thoms C. Gattinger A. Jacob M. Thomas F. M. Gleixner G. (2010). Direct and indirect effects of tree diversity drive soil microbial diversity in temperate deciduous forest. Soil Biol. Biochem. 42, 1558–1565. 10.1016/j.soilbio.2010.05.030

[ref125] Tkacz A. Poole P. (2015). Role of root microbiota in plant productivity. J. Exp. Bot. 66, 2167–2175. 10.1093/jxb/erv157, PMID: 25908654 PMC4986727

[ref126] Townsend A. R. Cleveland C. C. Houlton B. Z. Alden C. B. White J. W. C. (2011). Multi-element regulation of the tropical forest carbon cycle. Front. Ecol. Environ. 9, 9–17. 10.1890/100047

[ref127] Ulrich A. Klimke G. Wirth S. (2008). Diversity and activity of cellulose-decomposing bacteria, isolated from a sandy and a loamy soil after long-term manure application. Microb. Ecol. 55, 512–522. 10.1007/s00248-007-9296-0, PMID: 17665240

[ref128] Urbanová M. Šnajdr J. Baldrian P. (2015). Composition of fungal and bacterial communities in forest litter and soil is largely determined by dominant trees. Soil Biol. Biochem. 84, 53–64. 10.1016/j.soilbio.2015.02.011

[ref129] Uroz S. Buée M. Deveau A. Mieszkin S. Martin F. (2016a). Ecology of the forest microbiome: highlights of temperate and boreal ecosystems. Soil Biol. Biochem. 103, 471–488. 10.1016/j.soilbio.2016.09.006

[ref130] Uroz S. Oger P. Lepleux C. Collignon C. Frey-Klett P. Turpault M. P. (2011). Bacterial weathering and its contribution to nutrient cycling in temperate forest ecosystems. Res. Microbiol. 162, 821–831. 10.1016/j.resmic.2011.01.013 21315149

[ref131] Uroz S. Oger P. Tisserand E. CéBron A. Turpault M.-P. Bueé M. (2016b). Specific impacts of beech and Norway spruce on the structure and diversity of the rhizosphere and soil microbial communities. Sci. Rep. 6, 1–11. 10.1038/srep27756 27302652 PMC4908602

[ref132] van den Heuvel R. N. van der Biezen E. Jetten M. S. M. Hefting M. M. Kartal B. (2010). Denitrification at pH 4 by a soil-derived Rhodanobacter-dominated community. Environ. Microbiol. 12, 3264–3271. 10.1111/j.1462-2920.2010.02301.x, PMID: 20649643

[ref133] Ventura M. Canchaya C. Tauch A. Chandra G. Fitzgerald G. F. Chater K. F.. (2007). Genomics of Actinobacteria: tracing the evolutionary history of an ancient phylum. Microbiol. Mol. Biol. Rev. 71, 495–548. 10.1128/MMBR.00005-07, PMID: 17804669 PMC2168647

[ref134] Vranova V. Rejsek K. Formanek P. (2013). Aliphatic, cyclic, and aromatic organic acids, vitamins, and carbohydrates in soil: a review. Sci. J 2013:524239. 10.1155/2013/524239, PMID: 24319374 PMC3844170

[ref135] Wang Y. Qian P. Y. (2009). Conservative fragments in bacterial 16S rRNA genes and primer design for 16S ribosomal DNA amplicons in metagenomic studies. PLoS One 4:e7401. 10.1371/journal.pone.0007401, PMID: 19816594 PMC2754607

[ref136] Wang W. Wang H. Feng Y. Wang L. Xiao X. Xi Y. (2016). Consistent responses of the microbial community structure to organic farming along the middle and lower reaches of the Yangtze River. Sci. Rep. 6:35046. 10.1038/srep35046 27725750 PMC5057158

[ref137] Wemheuer B. Wemheuer F. Daniel R. (2012). RNA-based assessment of diversity and composition of active archaeal communities in the German bight. Archaea 2012:695826. 10.1155/2012/695826, PMID: 23197941 PMC3502831

[ref138] Wemheuer B. Wemheuer F. Meier D. Billerbeck S. Giebel H.-A. Simon M.. (2017). Linking compositional and functional predictions to decipher the biogeochemical significance in DFAA turnover of abundant bacterioplankton lineages in the North Sea. Microorganisms 5:68. 10.3390/microorganisms5040068, PMID: 29113091 PMC5748577

[ref139] Wickham H. (2016). ggplot2: Elegant graphics for data analysis. (New York: Springer-Verlag) Available at: http://ggplot2.org

[ref140] Wood S. A. Gilbert J. A. Leff J. W. Fierer N. D’Angelo H. Bateman C. (2017). Consequences of tropical forest conversion to oil palm on soil bacterial community and network structure. Soil Biol. Biochem. 112, 258–268. 10.1016/j.soilbio.2017.05.019

[ref141] Yokobe T. Hyodo F. Tokuchi N. (2018). Seasonal effects on microbial community structure and nitrogen dynamics in temperate forest soil. Forests 9, 153–169. 10.3390/f9030153

[ref142] Yousuf B. Kumar R. Mishra A. Jha B. (2014a). Differential distribution and abundance of diazotrophic bacterial communities across different soil niches using a gene-targeted clone library approach. FEMS Microbiol. Lett. 360, 117–125. 10.1111/1574-6968.12593 25196726

[ref143] Yousuf B. Kumar R. Mishra A. Jha B. (2014b). Unravelling the carbon and sulphur metabolism in coastal soil ecosystems using comparative cultivation-independent genome-level characterisation of microbial communities. PLoS One 9:107025. 10.1371/journal.pone.0107025 PMC4167329 25225969

[ref144] Zhalnina K. Louie K. B. Hao Z. Mansoori N. Nunes da Rocha U. Shi S. (2018). Dynamic root exudate chemistry and microbial substrate preferences drive patterns in rhizosphere microbial community assembly. Nat. Microbiol. 4, 470–480. 10.1038/s41564-018-0129-3 29556109

[ref145] Žifčáková L. Větrovský T. Howe A. Baldrian P. (2016). Microbial activity in forest soil reflects the changes in ecosystem properties between summer and winter. Environ. Microbiol. 18, 288–301. 10.1111/1462-2920.13026, PMID: 26286355

[ref146] Žifčáková L. Větrovský T. Lombard V. Henrissat B. Howe A. Baldrian P. (2017). Feed in summer, rest in winter: microbial carbon utilization in forest topsoil. Microbiome 5:122. 10.1186/s40168-017-0340-0 28923122 PMC5604414

