# Supplementary material for: Tree Species Shape Soil Bacterial Community Structure and Function in Temperate Deciduous Forests
Source: Front Microbiol. 2019 Jul 9;10:1519. doi: 10.3389/fmicb.2019.01519 (PMC6629791; doi:10.3389/fmicb.2019.01519)
Supplement: Supplementary file 1 [file Data_Sheet_1.PDF]

Supplementary Material

Tree species shape soil bacterial community structure and function in temperate deciduous forests

Amélie Dukunde[1], Dominik Schneider[1], Marcus Schmidt[2], Edzo Veldkamp[2], Rolf Daniel[1]*

[1]Department of Genomic and Applied Microbiology, Institute of Microbiology and Genetics, Georg-August University of Göttingen, Grisebachstraße 8, 37077, Göttingen, Germany

[2]Büsgen Institute – Soil Science of Tropical and Subtropical Ecosystems, Georg-August University of Göttingen, Büsgenweg 2, 37077 Göttingen, Germany

Content

Figure S1. Boxplots showing soil environmental parameters in mono and mixed stands.

Figure S2. Alpha diversity metrics of soil bacterial OTUs across different tree stands of the entire dataset.

Figure S3. Alpha diversity metrics for DNA-derived and RNA-derived OTUs, across different tree stands.

Figure S4.| Bacterial orders at total community level showing significant change in relative abundance across different tree stands.

Figure S5. Bacterial orders at potentially active community level showing significant change in relative abundance across different tree stands

Figure S6.| Bipartite association networks between soil bacterial communities (genus level) and tree stands.

Figure S7. Non-metric multidimensional scaling (NMDS) of potential gene functions in methane metabolism

Table S1. Summary of sequence data generated with high throughput pyrotag sequencing.

Table S2. Statistical tests of tree stand effects on bacterial community.

Table S3. Summary of abundant soil bacterial genera across mono and mixed stands at entire community level.

Table S4. Summary of abundant soil bacterial genera across mono and mixed stands at potentially active community level.

Table S5. Statistical tests of tree stand effects on bacterial community function.

Table S6. List of selected genes associated with energy metabolism.

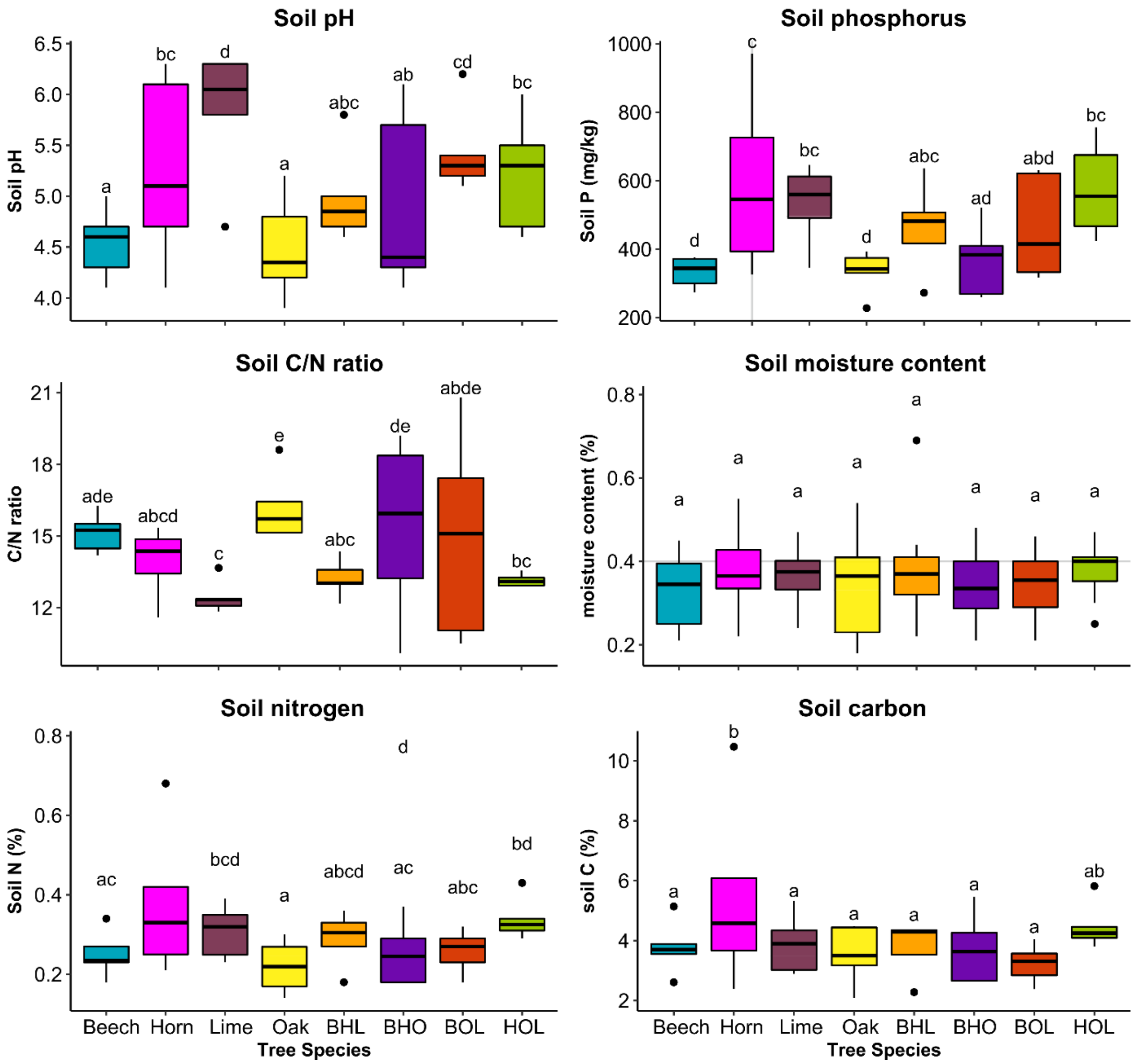

Supplementary Figure S1.| Boxplots showing soil environmental parameters in mono and mixed stands. Mean values (n = 6 replicated stand) are given. Horizontal bars show median value. Mean values with similar letters determined by ANOVA with Tukey HSD post-hoc test shared significant similarities (p 0.05). Soil data was previously collected and described by Schmidt et al. (2015). Abbreviations: BHL, beech, hornbeam, lime; BHO, beech, hornbeam, oak; BOL, beech, oak, lime; HOL, hornbeam, oak, lime.

Reference:

Schmidt, M., Veldkamp, E., and Corre, M. D. (2015). Tree species diversity effects on productivity, soil nutrient availability and nutrient response efficiency in a temperate deciduous forest. *For. Ecol. Manage.* 338, 114–123. doi:10.1016/j.foreco.2014.11.021

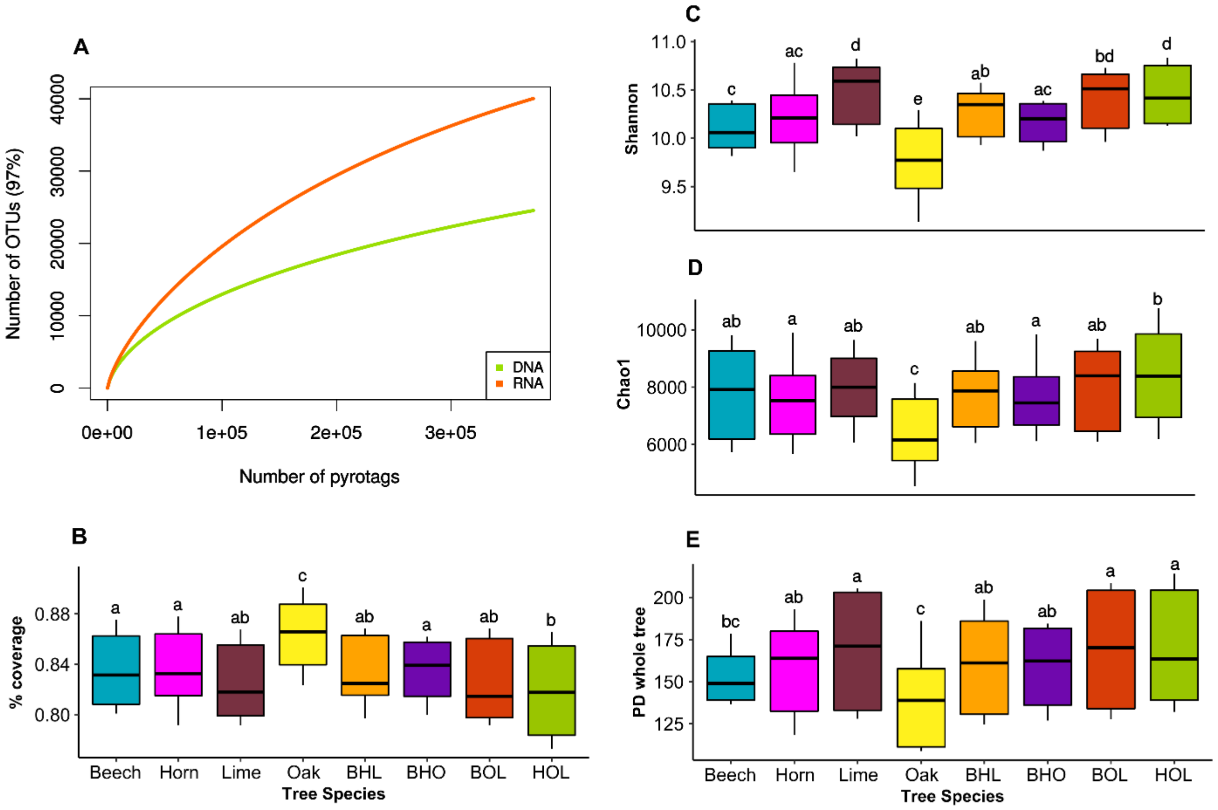

Supplementary Figure S2.| Alpha diversity metrics of soil bacterial OTUs across different tree stands of the entire dataset. **(A)** Rarefaction curves of subsampled OTUs for environmental DNA and RNA, at 97% similarity. **(B)** Good's coverage, **(C)** Shannon richness index, **(D)** Chao1 estimator and **(E)** phylogenetic diversity (PD). Mean values are given (n = 6). Horizontal bars represent the median value. Mean values with similar letters determined by ANOVA with Tukey HSD post-hoc test shared significant similarities ($p \leq 0.05$). Abbreviations: BHL, beech, hornbeam, lime; BHO, beech, hornbeam, oak; BOL, beech, oak, lime; HOL, hornbeam, oak, lime.

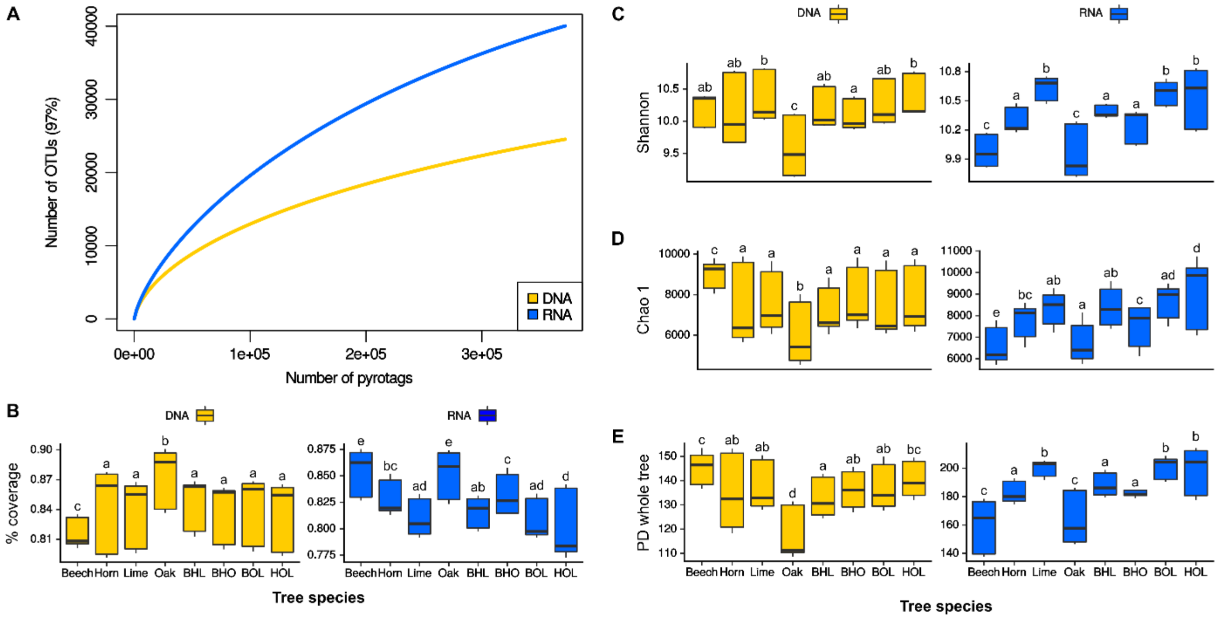

Supplementary Figure S3. Alpha diversity metrics for DNA-derived and RNA-derived OTUs, across different tree stands. **(A)** Rarefaction curves of subsampled OTUs for environmental DNA and RNA, at 97% similarity. **(B)** Good's coverage, **(C)** Shannon richness index, **(D)** Chao1 estimator and **(E)** phylogenetic diversity (PD). Mean values are given (n = 6). Horizontal bars represent the median value. Mean values with similar letters determined by ANOVA with Tukey HSD post-hoc test shared significant similarities ($p \leq 0.05$). Abbreviations: BHL, beech, hornbeam, lime; BHO, beech, hornbeam, oak; BOL, beech, oak, lime; HOL, hornbeam, oak, lime.

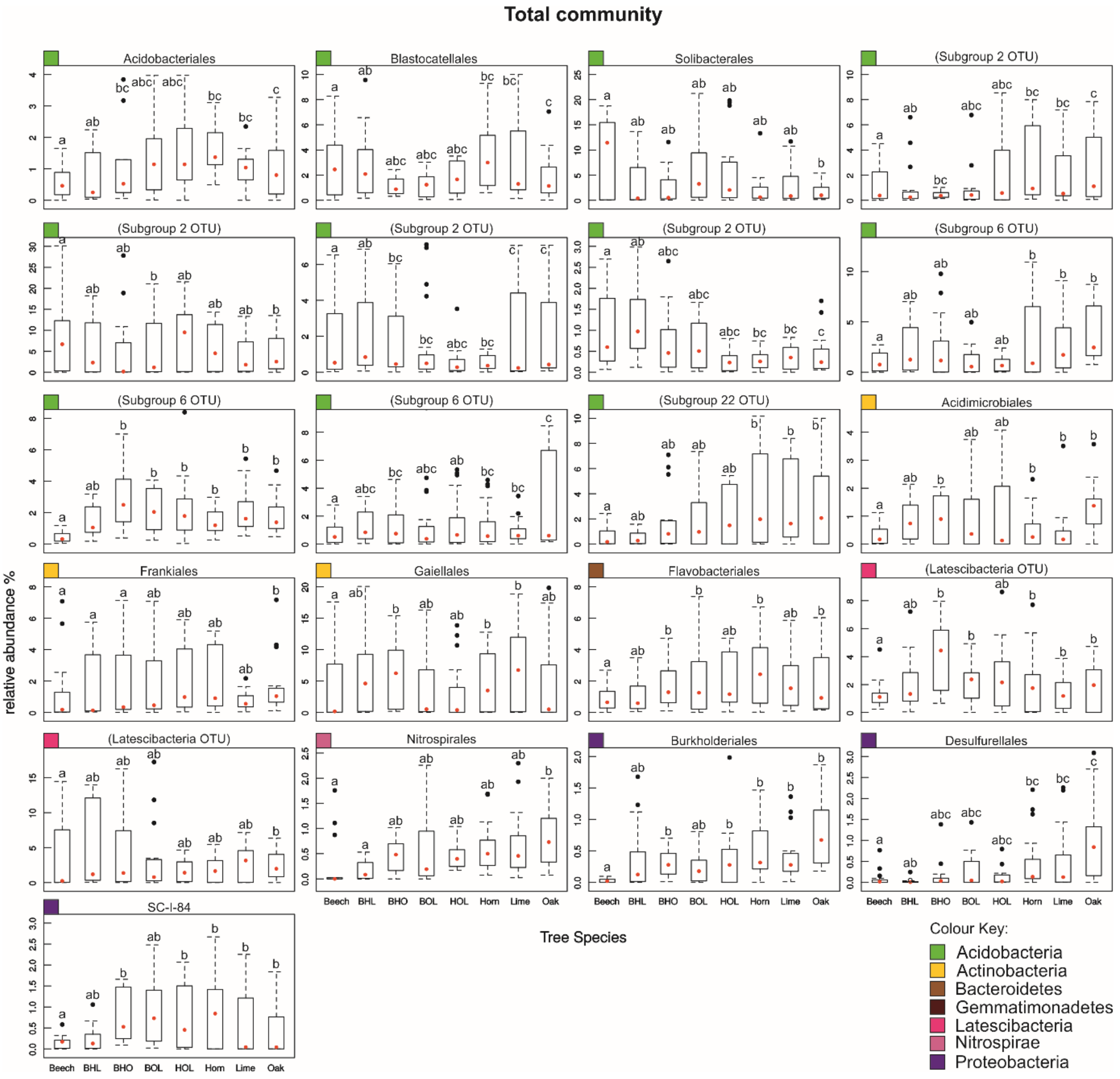

Supplementary Figure S4.| Bacterial orders at total community level showing significant change in relative abundance across different tree stands. Only bacteria orders belonging to dominant phyla (relative abundance higher than 1 %) are shown. In the case that the order could not be assigned, the taxonomic name at the highest determined taxonomic resolution is given in parenthesis. Mean values of replicate stands (*n* = 6) for three seasons are given. Statistical calculations employed Kruskal-Wallis test with Dunn's post hoc test. A $p \leq 0.05$ was considered statistically significant. Mean values with similar letters determined by ANOVA with Tukey HSD post-hoc test shared significant similarities ($p \leq 0.05$). Red dots represent the median value. Black dots represent outlying data. Abbreviations: BHL, beech, hornbeam, lime; BHO, beech, hornbeam, oak; BOL, beech, oak, lime; HOL, hornbeam, oak, lime.

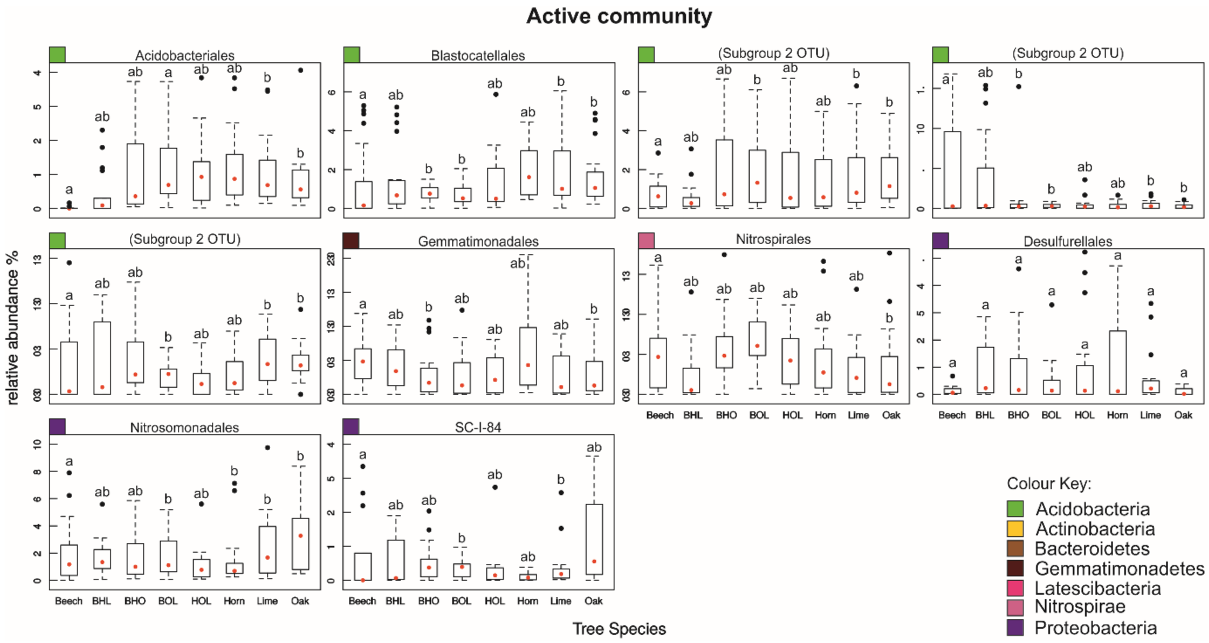

Supplementary Figure S5. Bacterial orders at potentially active community level showing significant change in relative abundance across different tree stands. Only bacteria orders belonging to dominant phyla (greater than 1 %) are shown. In the case that the order could not be assigned, the taxonomic name at the highest determined taxonomic resolution is given in parenthesis. Mean values of replicate stands (n = 6) for three seasons are given. Statistical calculations employed Kruskal-Wallis test with Dunn's post hoc test. Mean values with similar letters determined by ANOVA with Tukey HSD post-hoc test share significant similarities ($p \leq 0.05$). Red dots represent the median value. Black dots represent outlying data. Abbreviations: BHL, beech, hornbeam, lime; BHO, beech, hornbeam, oak; BOL, beech, oak, lime; HOL, hornbeam, oak, lime.

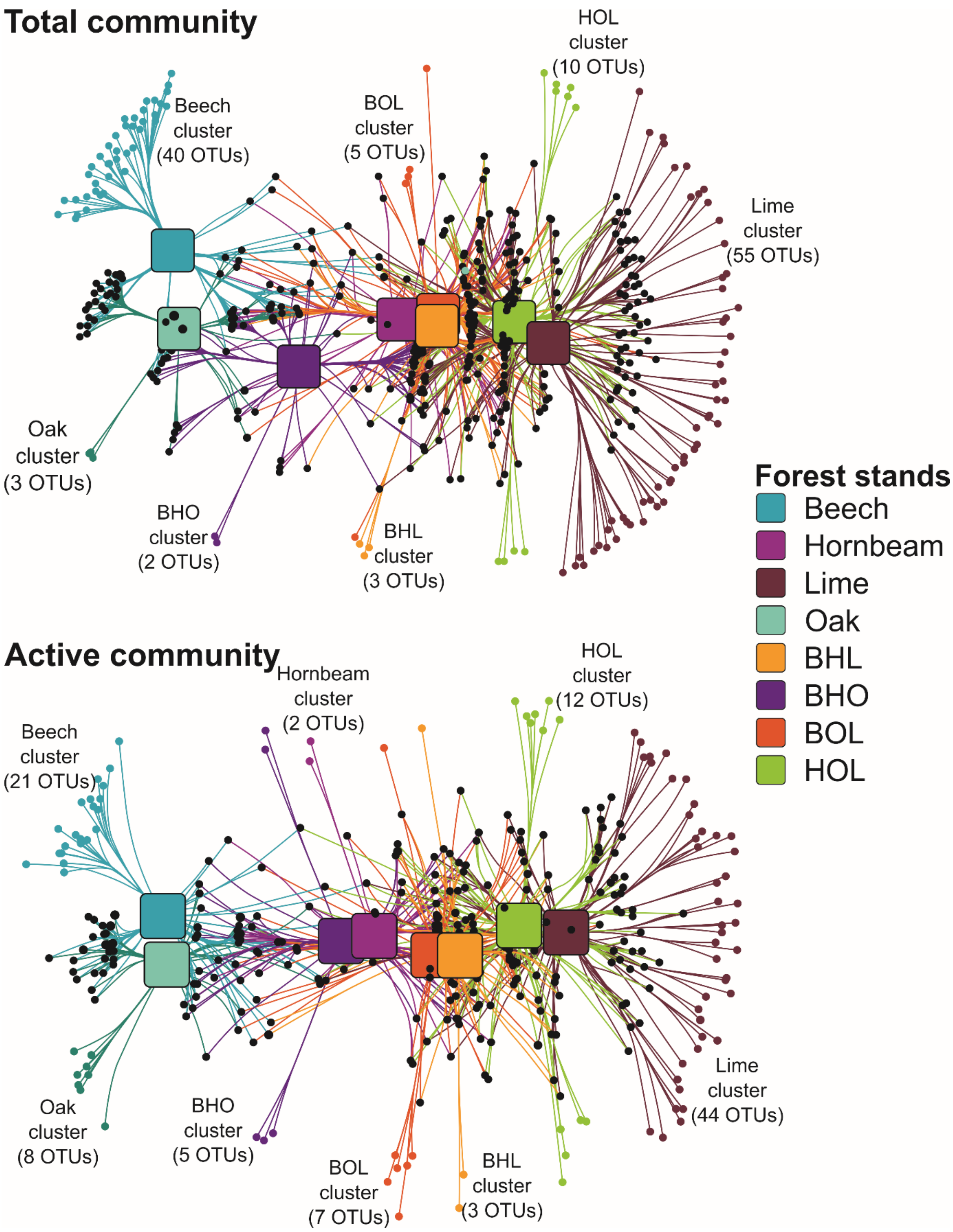

Supplementary Figure S6.| Bipartite association networks between soil bacterial communities (genus level) and tree stands. Source nodes (rounded squares) represent tree stands and edges represent associations between stands and bacterial OTUs (circles, target nodes). Edges are coloured according to the source tree stand and the length of edges is weighted according to association strength. Unique clusters, which associate with one tree species, consist of nodes coloured as the corresponding stand. Numbers of OTUs making up respective unique clusters are given in parenthesis. Black circles represent OTUs with significant cross association between two or more stands. Target node sizes represent mean relative abundance of OTUs across all mono plots. Data only represents OTUs that showed significant positive association with tree stands ($p \leq 0.05$). For visualisation, edges were bundled with a stress value of 3.

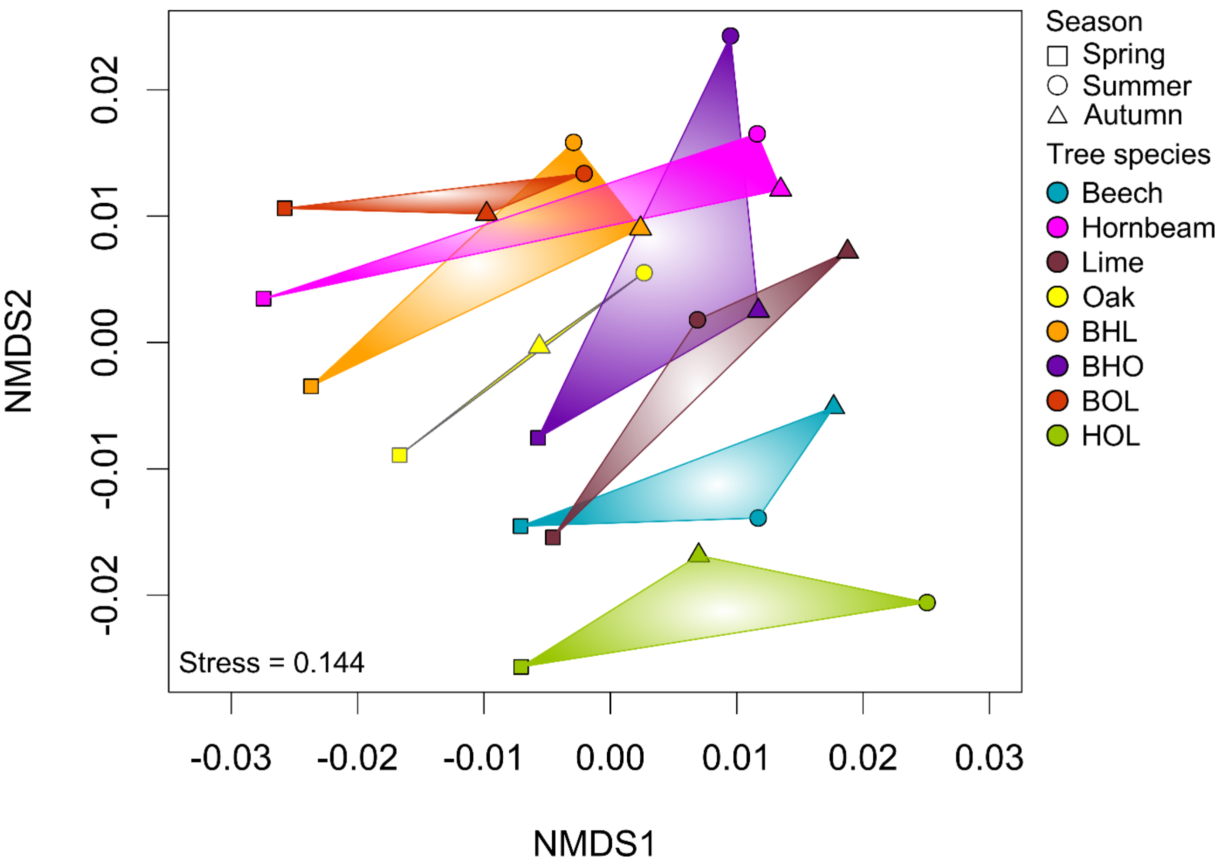

Supplementary Figure S7.| Non-metric multidimensional scaling (NMDS) of potential gene functions in methane metabolism. Significant grouping of metabolic genes according to tree species (*p* 0.033) is given. NMDS was constructed with a Bray Curtis distance matrix using KEGG orthologs predicted with Tax4fun (Aßhauer et al., 2015). Genes used to construct the plot are listed in Supplementary Table S5.

Reference:

Aßhauer, K. P., Wemheuer, B., Daniel, R., and Meinicke, P. (2015). Tax4Fun: Predicting functional profiles from metagenomic 16S rRNA data. *Bioinformatics* 31, 2882–2884. doi:10.1093/bioinformatics/btv287

Supplementary Table S1. Summary of sequence data generated with high throughput pyrotag sequencing.

**Reads after key steps of sequencing pipeline**	**Template**	
	DNA	RNA[a]
1. Raw reads	3,335,821	1,308,705
2. Quality filtering	3,052,200	1,291,122
3. Chimera and singleton removal	2,402,787	857,868
4. Taxonomy filtering	2,401,078	857,464
% Recovery	72.4	65.7
Total OTUs[b]	**40,385**	**52,277**

[a] Reads for RNA were generated via cDNA
[b] OTUs generated after clustering of abovementioned reads. Numbers represent total count across all plots in spring, summer and autumn from subsampled datasets of environmental DNA and RNA.
[c] Recovery refers to the percentage of sequence reads after processing over the total reads.

Supplementary Table S2. Statistical tests of tree stand effects on bacterial community structure. Results of ANOSIM and PERMANOVA were calculated using weighted UniFrac matrices representing the total (DNA-based) and potentially active (RNA-based) bacterial community. A *p* value ≤ 0.05 was considered significant.

Test	ANOSIM				PERMANOVA			
	DNA		RNA		DNA		RNA	
	p-value	R	*p*-value	R	*p*-value	Pseudo F	*p*-value	Pseudo F
Tree species	0.001	0.72	0.001	0.63	0.001	9.4	0.001	4.9
Stand type	0.008	0.22	0.002	0.23	0.1	2.4	0.044	2.9
Season	0.464	-0.009	0.038	0.132	0.529	0.73	0.132	1.7

NMDS goodness of fit			
	p value	R^2	Significance
Template	0.001	0.439	***
Tree species	0.001	0.506	***
Stand type	0.043	0.068	*
Season	0.858	0.014	NS

Environmental parameters				Mantel test		
	p value	R^2	Significance	*p* value	R^2	Significance
pH	0.001	0.809	***	0.001	0.518	***
N	0.001	0.353	***	0.001	0.215	***
C/N ratio	0.001	0.683	***	0.001	0.384	***
P	0.001	0.589	***	0.001	0.418	***
MC	0.001	0.655	***	0.001	0.430	***
C	0.490	0.341	NS	0.936	-0.079	NS

Abbreviations: N, nitrogen; C/N, carbon/nitrogen; P, phosphorus; MC, moisture content; C, carbon

Significance 0 = ***, 0.001 = **, 0.01 = *, 0.05 = *, greater than 0.05 = not significant (NS)

Supplementary Table S3. Summary of abundant soil bacterial genera across mono and mixed stands at entire community level. Values represent mean relative abundance for 6 replicate stands in spring, summer and autumn. Values are given for total (DNA-based) community. Only genera from abundant orders (greater than 1 %) are given. In the case that the order or genus could not be assigned, the taxonomic name at the highest determined taxonomic resolution is given in parenthesis.

**Total community**		**Relative abundance (%) in forest stands**								
Order	Genus	Mono stands				Mixed stands				
		**Beech**	**Horn**	**Lime**	**Oak**	**BHL**	**BHO**	**BOL**	**HOL**	**Average**
Acidobacteria										
Acidobacteriales	*(Acidobacteriaceae)*	13.74	6.87	1.17	12.84	5.24	9.31	5.11	3.32	7.20
Solibacterales	*Bryobacter*	6.20	4.34	1.52	6.90	3.57	5.28	4.11	2.78	4.34
	Candidatus Solibacter	5.05	3.85	2.61	4.30	4.09	4.20	3.61	3.18	3.86
Subgroup 2	*(Subgroup 2)*	13.20	6.57	1.29	11.99	4.28	7.67	4.48	3.39	6.61
Subgroup 6	*(Subgroup 6)*	2.11	4.35	8.01	2.10	5.55	3.49	4.92	5.85	4.55
	(Subgroup 6)	0.86	2.06	3.41	0.85	2.68	1.50	2.22	2.62	2.02
Actinobacteria										
Acidimicrobiales	*(Acidimicrobiales)*	0.77	0.99	0.95	0.83	0.93	0.93	1.02	1.07	0.94
Frankiales	*Acidothermus*	2.53	4.03	0.91	3.02	2.21	3.19	2.25	1.95	2.51
Gaiellales	*(Gaiellales)*	1.15	2.24	2.14	1.08	2.30	1.78	2.47	2.62	1.97
Solirubrobacterales	*(Solirubrobacterales)*	0.17	0.48	0.71	0.17	0.48	0.39	0.59	0.70	0.46
Bacteroidetes										
Cytophagales	*(Cytophagaceae)*	0.66	1.32	1.58	0.94	1.46	1.33	1.28	1.80	1.29
Flavobacteriales	*Flavobacterium*	0.33	1.02	1.79	0.45	0.81	0.73	1.12	1.69	0.99
Sphingobactriales	*(Chitinophagaceae)*	0.97	1.01	0.89	1.04	0.97	1.06	1.09	1.04	1.01
Gemmatimonadetes										
Gemmatimonadales	*Gemmatimonas*	1.46	1.19	1.58	1.09	1.61	1.10	1.89	1.43	1.42
	(Gemmatimonadaceae)	1.25	1.10	1.34	0.71	1.15	1.02	1.49	1.17	1.15
Latescibacteria										
(Latescibacteria)	*(Latescibacteria)*	0.20	0.62	1.48	0.16	0.90	0.58	0.81	0.89	0.70,
	(Latescibacteria)	0.07	0.22	0.42	0.09	0.45	0.13	0.34	0.46	0.27
	(Latescibacteria)	0.02	0.09	0.27	0.03	0.15	0.06	0.12	0.11	0.11
Nitrospirae										
Nitrorpirales	*(Nitrospirales)*	0.04	0.44	1.38	0.06	0.62	0.35	0.56	0.62	0.51
	Nitrospira	0.35	0.26	0.51	0.20	0.30	0.19	0.37	0.30	0.31
Proteobacteria										
Burkholderiales	*(Comamonadaceae)*	0.31	0.57	0.92	0.37	0.74	0.47	0.68	0.93	0.62
	Variovorax	0.10	0.20	0.27	0.13	0.23	0.14	0.26	0.26	0.20

1	*Paraburkholderia*	0.15	0.08	0.11	0.13	0.05	0.14	0.05	0.04	0.09
	Rhizobacter	0.03	0.07	0.14	0.03	0.11	0.06	0.09	0.14	0.08
Desulfurellales	*(Desulfurellaceae)*	0.29	0.54	1.33	0.25	0.74	0.47	0.77	0.86	0.66
	(Desulfurellaceae)	0.00	0.02	0.06	0.00	0.02	0.01	0.02	0.04	0.02
Myxococcales	*Haliangium*	0.85	1.38	1.38	1.17	1.41	1.30	1.72	1.51	1.34
	Sorangium	0.60	0.50	0.35	0.58	0.45	0.49	0.55	0.38	0.49
Nitrosomonadales	*(Nitrosomonadaceae)*	1.30	1.78	2.64	1.21	2.19	1.95	2.40	2.05	1.95
	Nitrosospira	0.00	0.01	0.01	0.00	0.01	0.01	0.01	0.01	0.01
Rhizobiales	*(Xanthobacteraceae)*	1.20	2.83	3.89	1.37	3.40	2.05	3.12	3.61	2.68
	Bradyrhizobium	1.79	2.57	2.45	2.19	2.74	2.61	2.52	2.83	2.46
	Rhizomicrobium	2.77	2.25	0.75	3.15	2.05	2.82	1.71	1.47	2.12
	Variibacter	1.67	1.80	1.51	1.48	1.82	1.52	1.75	1.76	1.66
Rhodospirillales	*(DA111)*	2.01	1.78	2.25	1.40	2.08	1.50	2.00	2.13	1.89
	(Acetobacteraceae)	1.97	1.67	0.86	2.55	1.67	2.33	1.20	1.02	1.66
	Reyranella	0.62	1.46	2.47	0.84	1.88	1.12	1.37	1.70	1.43
Xanthomonadales	*Rhodanobacter*	1.87	1.87	1.09	2.17	1.83	1.54	2.68	1.85	1.86
	Acidibacter	1.58	1.37	1.13	1.72	1.07	1.46	1.03	0.94	1.29
	(Xanthomonadales)	1.58	1.32	0.19	1.20	0.78	2.00	0.69	0.54	1.13

Abbreviations: BHL, beech, hornbeam, lime; BHO, beech, hornbeam, oak; BOL, beech, oak, lime; HOL, hornbeam, oak, lime

Supplementary Table S4. Summary of abundant soil bacterial genera across mono and mixed stands at potentially active community level. Values represent mean relative abundance for 6 replicate stands in spring, summer and autumn. Values are given for the potentially active (RNA-based) community. Only genera from abundant orders (greater than 1 %) are given. In the case the order or genus could not be assigned, the taxonomic name at the highest determined taxonomic resolution is given in parenthesis.

**Active Community**		**Forest stands**								
		Mono stands				Mixed stands				
Order	Genus	**Beech**	**Horn**	**Lime**	**Oak**	**BHL**	**BHO**	**BOL**	**HOL**	**Average**
Acidobacteria										
Acidobacteriales	*(Acidobacteriaceae)*	8.29	4.07	0.70	9.53	3.44	5.45	3.21	1.88	4.57
Solibacterales	*Candidatus Solibacter*	6.81	3.45	2.09	6.47	2.69	3.56	3.73	2.48	3.91
	Bryobacter	5.89	4.21	1.00	7.00	2.72	3.44	3.13	1.53	3.62
Subgroup 2	*(Subgroup 2)*	7.57	3.77	0.84	6.74	3.16	3.82	2.37	1.72	3.749
Subgroup 6	*(Subgroup 6)*	0.88	1.56	3.46	0.73	1.92	1.62	2.01	2.26	1.80
	(Subgroup 6)	0.34	0.63	1.43	0.27	0.86	0.75	0.90	1.07	0.78
Actinobacteria										
Acidimicrobiales	*(Acidimicrobiales)*	0.79	0.75	0.858	0.82	0.74	0.84	0.75	0.70	0.78
Frankiales	*Acidothermus*	4.51	5.56	1.189	5.04	3.74	5.69	3.11	2.90	3.97
Gaiellales	*(Gaiellales)*	0.65	0.91	0.897	0.59	1.01	0.83	0.87	1.05	0.85
Solirubrobacterales	*(Solirubrobacterales)*	0.05	0.15	0.280	0.03	0.17	0.10	0.12	0.20	0.14
Bacteroidetes										
Cytophagales	*(Cytophagaceae)*	0.18	0.23	0.55	0.16	0.40	0.49	0.36	0.53	0.36
Flavobacteriales	*Flavobacterium*	0.24	1.11	1.79	0.32	0.88	0.83	2.72	2.23	1.26
Sphingobactriales	*(Chitinophagaceae)*	1.89	2.48	2.75	2.17	2.35	2.15	3.21	2.88	2.49
Gemmatimonadetes										
Gemmatimonadales	*Gemmatimonas*	0.48	0.31	0.46	0.34	0.55	0.28	0.46	0.43	0.41
	(Gemmatimonadaceae)	0.31	0.22	0.40	0.13	0.25	0.22	0.25	0.28	0.26
Latescibacteria										
(Latescibacteria)	*(Latescibacteria)*	0.06	0.10	0.36	0.02	0.16	0.15	0.19	0.22	0.16
	(Latescibacteria)	0.04	0.08	0.18	0.03	0.17	0.08	0.18	0.24	0.12
	(Latescibacteria)	0.01	0.00	0.03	0.01	0.00	0.02	0.01	0.02	0.01
Nitrospirae										
Nitrorpirales	*(Nitrospirales)*	0.19	0.09	0.17	0.08	0.12	0.12	0.13	0.10	0.12
	Nitrospira	0.01	0.06	0.26	0.00	0.06	0.09	0.07	0.13	0.09
Proteobacteria										
Burkholderiales	*(Comamonadaceae)*	0.81	1.68	2.30	0.87	2.44	1.40	2.06	2.38	1.74
	Variovorax	0.38	0.57	0.82	0.40	0.70	0.41	0.75	0.95	0.62

	Rhizobacter	0.10	0.25	0.57	0.13	0.45	0.26	0.43	0.57	0.34
Desulfurellales	*(Desulfurellaceae)*	0.37	0.67	1.06	0.26	0.66	0.53	0.60	0.73	0.61
	(Desulfurellaceae)	0.00	0.04	0.16	0.00	0.06	0.04	0.05	0.08	0.06
Myxococcales	*Haliangium*	3.70	5.39	8.29	3.92	7.48	5.18	6.65	7.35	6.00
	Sorangium	3.66	2.59	2.43	3.06	2.70	2.27	2.44	2.10	2.66
Nitrosomonadales	*(Nitrosomonadaceae)*	0.95	1.23	1.99	0.81	1.70	1.66	1.88	1.49	1.46
	Nitrosospira	0.02	0.03	0.04	0.01	0.06	0.04	0.06	0.07	0.04
Rhizobiales	*(Xanthobacteraceae)*	2.96	5.33	5.73	2.54	5.98	4.40	5.46	5.89	4.79
	Variibacter	3.28	4.02	2.81	2.54	3.42	3.78	3.20	3.35	3.30
	Bradyrhizobium	2.46	3.51	3.07	2.27	3.30	3.29	2.75	3.98	3.17
	Rhizomicrobium	1.21	0.90	0.26	1.37	0.67	0.82	0.98	0.42	0.83
Rhodospirillales	*(DA111)*	4.05	3.11	2.94	2.78	3.74	2.82	2.86	2.88	3.15
	(Acetobacteraceae)	2.59	2.16	0.74	3.07	1.67	2.57	1.39	0.71	1.86
	Reyranella	0.44	0.67	0.72	0.50	0.63	0.47	0.74	0.57	0.59
Xanthomonadales	*Rhodanobacter*	1.90	1.32	0.06	2.81	0.94	2.32	0.75	0.24	1.29
	Acidibacter	1.42	1.43	0.93	1.93	1.04	1.51	0.98	0.91	1.27
	(Xanthomonadales)	1.01	1.15	0.47	1.23	0.68	1.10	0.71	0.56	0.87

Abbreviations: BHL, beech, hornbeam, lime; BHO, beech, hornbeam, oak; BOL, beech, oak, lime; HOL, hornbeam, oak, lime

Supplementary Table S5. Statistical tests of tree stand effects on bacterial community function. NMDS was calculated using Bray Curtis distance matrices representing the genes predicted from the potentially active (RNA-based) bacterial community with Tax4Fun (Aßhauer et al., 2015). Tax4Fun predicted assigned function to 45% of input OTUs from the potentially active community. A *p* value ≤ 0.05 was considered statistically significant.

NMDS goodness of fit	*p*-value	R^2	Significance code
**Carbon metabolism**			
Tree species	0.079	0.45	NS
Stand type	0.421	0.041	NS
Season	0.001	0.42	***
**Methane metabolism**			
Tree species	0.033	0.48	*
Stand type	0.763	0.011	NS
Season	0.001	0.39	***
**Sulphur metabolism**			
Tree species	0.078	0.44	NS
Stand type	0.239	0.065	NS
Season	0.001	0.42	***
**Nitrogen metabolism**			
Tree species	0.203	0.38	NS
Stand type	0.398	0.04	NS
Season	0.001	0.49	***

Significance 0 = ***, 0.001 = **, 0.01 = *, 0.05 = *, greater than 0.1 = not significant (NS)

Reference:

Aßhauer, K. P., Wemheuer, B., Daniel, R., and Meinicke, P. (2015). Tax4Fun: Predicting functional profiles from metagenomic 16S rRNA data. *Bioinformatics* 31, 2882–2884. doi:10.1093/bioinformatics/btv287

Supplementary Table S6. List of selected genes associated with energy metabolism. Gene functions were predicted from the potentially active (RNA-based) bacterial community with Tax4Fun (Aßhauer et al., 2015).

KEGG ortholog	Name	KEGG ortholog	Name
**Calvin Cycle**		**Hemicellulases**	
K00174 - K00177	2-oxoglutarate ferredoxin oxidoreductase	K01181	endo-1,4-beta-xylanase
K01601 - K01602	ribulose-bisphosphate carboxylase	K01198	xylan 1,4-beta-xylosidase
K00239 - K00242	succinate dehydrogenase	K01218	mannan endo-1,4-beta-mannosidase
**Methane metabolism**		K01224	arabinogalactan endo-1,4-beta-galactosidase
K14028 - K14029	methanol dehydrogenase (cytochrome c)	K01684	galactonate dehydratase
K00190 - K00198	carbon monoxide dehydrogenase / acetyl-CoA synthase	K01811	alpha-D-xyloside xylohydrolase
K03518 - K03520	carbon-monoxide dehydrogenase small subunit	K15531	oligosaccharide reducing-end xylanase
K16154 - K16162	methane monooxygenase	K15921	arabinoxylan arabinofuranohydrolase
**Sulphur metabolism**		K15924	glucuronoarabinoxylan endo-1,4-beta-xylanase
K00380 - K00381	sulfite reductase (NADPH) flavoprotein	**Cellulases**	
K00390	phosphoadenosine phosphosulfate reductase	K01179	endoglucanase
K00392	sulfite reductase (ferredoxin)	K01180	endo-1,3(4)-beta-glucanase
K00394 - K00395	adenylylsulfate reductase	K01182	oligo-1,6-glucosidase
K00860	adenylylsulfate kinase	K01195	beta-glucuronidase
K00955	bifunctional enzyme CysN/CysC	K01225	cellulose 1,4-beta-cellobiosidase
K00956 - K00958	sulfate adenylyltransferase	K05349	beta-glucosidase
K11180 - K11181	sulfite reductase, dissimilatory-type	K05350	beta-glucosidase
**Nitrogen metabolism**		K16213	mannobiose 2-epimerase
K00360 - K00363	nitrate reductase (NADH)	**Chitinases**	
K00366 - K00367	ferredoxin-nitrite reductase	K01183	chitinase
K00368 - K00374	nitrite reductase (NO-forming)	K01452	chitin deacetylase
K00376	nitrous-oxide reductase	K03791	putative chitinase
K00531	nitrogenase	K03933	chitin-binding protein
K01428 - K01430	urease	K13381	bifunctional chitinase/lysozyme
K02164, K02305, K02448	nitric oxide reductase NorE, NorD	**Phosphatases**	
K02586 - K02588	nitrogenase molybdenum-iron protein (NifE, NifH)	K01077	alkaline phosphatase
K02591 - K02596, K02597	nitrogenase molybdenum-iron protein (NifN, NifT, NifV, NifX, NifZ)	K01078	acid phosphatase
K03385	cytochrome c-552	K01093	4-phytase / acid phosphatase
K04561	nitric oxide reductase subunit B	K01113	alkaline phosphatase D
K04747 - K04748	nitric oxide reductase protein (NorF, NorQ)	K03788	acid phosphatase (class B)
K10535	hydroxylamine oxidase	K09474	acid phosphatase (class A)
K10944 - K10946	ammonia monooxygenase (AmoABC)		
K15864	nitrite reductase (NO-forming) / hydroxylamine reductase		
K15876	cytochrome c-type protein		

Reference:

Aßhauer, K. P., Wemheuer, B., Daniel, R., and Meinicke, P. (2015). Tax4Fun: Predicting functional profiles from metagenomic 16S rRNA data. *Bioinformatics* 31, 2882–2884. doi:10.1093/bioinformatics/btv287
